# Safety of Repeated Administration of Xenogeneic Human Apoptotic State (Allocetra-OTS) in Sprague Dawley Rats

**DOI:** 10.3390/pharmaceutics16030426

**Published:** 2024-03-20

**Authors:** Chen Ankri, Oren Hershkovitz, Liat Hershkovitz, Meital Brami, Ronnie Levy, Hadar Sarig, Einat Souli, Barak Reicher, Veronique Amor-Baroukh, Dror Mevorach, Abraham Nyska

**Affiliations:** 1 Enlivex Therapeutics Ltd., Ness Ziona 7403618, Israel; chen@enlivexpharm.com (C.A.); oren@enlivexpharm.com (O.H.); barak@enlivexpharm.com (B.R.); veronique@enlivexpharm.com (V.A.-B.); mevorachd@enlivexpharm.com (D.M.); 2 Independent Researcher, Revadim 798200, Israel; liatorenh@gmail.com; 3 Pharmaseed Ltd., Ness Ziona 7404709, Israel; meital@pharmaseedltd.com (M.B.); ronnie@pharmaseedltd.com (R.L.); hadars@pharmaseedltd.com (H.S.); einat@pharmaseedltd.com (E.S.); 4 Sackler School of Medicine, Tel Aviv University, Tel Aviv 6139001, Israel

**Keywords:** cell-based therapies, human apoptotic cells, PBMC, safety

## Abstract

Apoptotic cells possess immunomodulatory effects that can be utilized to treat imbalanced immune conditions. Information on the preclinical safety of such treatment is sparse. In this study, the safety of apoptotic cells (Allocetra-OTS) was assessed in a GLP toxicological study on Sprague Dawley rats. Three doses of Allocetra-OTS or vehicle were administered intravenously (IV) for 3 consecutive days. Animals in the main study were sacrificed on day 4, while animals from the recovery groups were kept for 14 or 28 days. Allocetra-OTS was well tolerated, and no adverse effects were observed in terms of body weight, clinical signs, food consumption, or ophthalmologic observation. Thus, the No Observed Adverse Effect Level (NOAEL) dose was determined as the highest dose administered. An observed elevation in immune cells was suspected to be due to Allocetra-OTS, similarly to other clinical chemistry parameters; however, it was resolved in the recovery phases. Splenomegaly and dose-related extramedullary hematopoiesis (EMH) in the red pulp were observed, with no adverse events, and were considered to be a normal and expected reaction following the IV administration of cell-based therapies. In conclusion, under the conditions of this study, Allocetra-OTS was concluded to be safe, further supporting its potential candidacy for clinical studies.

## 1. Introduction

The therapeutic use of apoptotic cells is an emerging modality and is currently being assessed in clinical studies [1,2,3]. Apoptotic cells exert immunomodulatory effects [4] by physically interacting with macrophages and dendritic cells and by secreting several paracrine factors; the clearance process of these cells drives an immune homeostasis state [1,5,6,7,8,9,10,11]. Apoptosis, or programmed cell death, is a natural and critical process in tissue homeostasis and results in the immediate removal of a dying cell by macrophages and dendritic cells (DCs). DCs are professional antigen-presenting cells (APCs) in mammals; they mature from highly phagocytic precursors in response to ‘danger’ signals that are present in damaged or infected tissue. At the same time, immature DCs are capable of the large-scale phagocytosis of apoptotic cells and mature alternatively into a homeostatic state [12]. In a similar way, macrophages may produce a large variety of inflammatory cytokines in response to danger signals. However, the removal of apoptotic cells often occurs by macrophages downregulated by NF-Kb [13] and inflammatory responses in macrophages [14,15]. This combined effect was shown to induce several changes and functional activities in the engulfing APCs involving the regulation of immune responses. The clearance of apoptotic cells allows immune homeostasis, which can lead to a non-inflammatory state for both macrophages and DCs and contribute to the maintenance of peripheral homeostasis and tolerance in some clinical settings [11,16]. Conversely, under certain conditions, such as the killing of tumor cells by specific cell death inducers, the recognition of apoptotic tumor cells can promote an immunogenic response and anti-tumor immunity. Complex factors, such as cell type, the conditions leading to apoptosis, the apoptotic stage of the cells, and others, may lead to various pro-homeostatic effects mediated by macrophages and other APCs. Thus, apoptotic cells may deliver complex information that regulates different immunological responses in a context-dependent manner. The cell type, the cause of cell death, the microenvironment, quantity, and other factors may result in different immunomodulating signals, thus allowing for the modulation of immune processes in different clinical settings [9]. As many as 3 × 10^8^ cells undergo apoptosis every hour in our bodies, and one of the primary “eat me” signals expressed by apoptotic cells is phosphatidylserine (PtdSer). Apoptotic cells themselves are major contributors to the “non-inflammatory” nature of the engulfment process, some by secreting thrombospondin-1 (TSP-1) or adenosine monophosphate and possibly other immune-modulating “calm-down” signals that interact with macrophages and DCs. Apoptotic cells also produce “find me” and “tolerate me” signals to attract and immune modulate macrophages and DCs that express specific receptors for some of these signals [17,18]. Among emerging cell-based therapies, the use of dying cells defined as apoptotic cells as novel therapeutic strategies appears to hold significant promise. These apoptotic-cell-based therapies may be limited not only to transplantation settings, but also to other inflammatory diseases. The immunomodulatory mechanisms associated with physiological apoptotic cell removal (called efferocytosis) were reported in different experimental models of inflammatory diseases or transplantation settings using apoptotic cell infusion [19,20,21,22,23,24]. Apoptotic cells have been suggested as a clinical therapeutic modality in a variety of immune-mediated disorders [10,25,26,27,28], including autoimmune and autoinflammatory conditions (e.g., arthritis [29], colitis [15]), graft rejection [23], post-ischemic injury, and conditions characterized by a cytokine storm, such as septic shock [30] and COVID-19 [31]. The properties of apoptotic cells may promote an immunogenic response and anti-tumor activity by restoring organ macrophage homeostasis [32].

Cell-based human products may contain cells that are autologous, or allogeneic, and may or may not include cells that have been genetically modified, or which may or may not be combined with a device, scaffold, or mesh. Cell-based human products are aimed at repairing, restoring, replacing, or regenerating the structure and function of a damaged organ in order to ameliorate or cure previously untreatable injury or disease. Although there has been an increase and rapid progression in recent years in the development of new cellular therapies, more particularly the development of apoptotic cells, for clinical use, very limited data are available regarding their toxicity and tolerability in preclinical studies, and few guidelines have been published to assist in the design of appropriate preclinical studies [33].

**Figure 1:**
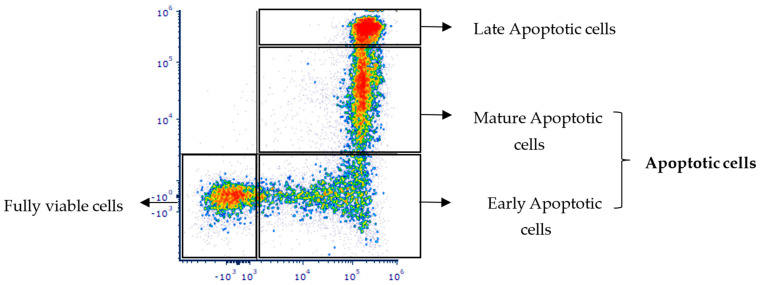
Apoptosis process analysis in Allocetra-OTS cells following annexin V and propidium iodide (PI) staining. Cells of Allocetra-OTS FDP were stained with annexin V (X axis) and propidium iodide (Y axis) to assess the prevalence of these apoptotic cells immediately following product thawing (representative Allocetra-OTS batch).

Enlivex Therapeutics Ltd. (Ness-Ziona, Israel) is developing Allocetra-OTS, an off-the-shelf macrophage reprogramming cell therapy produced by the induction and stabilization of apoptotic cells derived from the peripheral blood mononuclear cells (PBMCs) of healthy donors. The stable apoptotic state of these cells is verified by staining with annexin V and propidium iodide, thus avoiding potential adverse effects from necrotic cells (Figure 1).

The robust manufacturing process of Allocetra-OTS enables the production of stable apoptotic cells with a predefined quality attribute profile. A comprehensive nonclinical Good Laboratory Practice (GLP) biodistribution and persistence study demonstrated that the Allocetra-OTS levels in the blood, lungs, liver, spleen, and additional organs peaked 10–60 min following the injection, with the rapid clearance of Allocetra-OTS cells from most of the organs within 24 h (Supplementary Figure S1). The Allocetra-OTS cell suspension is transfused intravenously (IV) for systemic indications such as sepsis and COVID-19, intraperitoneally (IP) for the treatment of peritoneal metastasis, and intra-articularly for the treatment of osteoarthritis. It has shown a favorable safety profile in solid tumors (ClinicalTrials.gov identifiers NCT05431907 and NCT05581719). In addition, Allocetra-OTS has shown promising safety and preliminary efficacy in phase 1 [3] and phase 2 studies of sepsis (NCT03925857 and NCT04612413, respectively), as well as in COVID-19 in humans (NCT04922957) [2]. In addition, a previously conducted preclinical toxicology study in rats (using the liquid formulation of the product) showed that Allocetra-OTS was well tolerated, and that no serious adverse effects were observed.

To extend the shelf life of Allocetra-OTS, Enlivex Therapeutics Ltd. has developed a new cryopreserved formulation for the product. Here we present a recently conducted toxicology study aimed at establishing the safety of repeated IV administrations of the newly formulated Allocetra-OTS in Sprague Dawley (SD) rats. Allocetra-OTS was shown to be safe and well tolerated, with a major finding of spleen enlargement. These data, by showing similar safety profiles for both formulations, strengthen the safety profile of Allocetra-OTS. This finding is expected to be of significant importance for future clinical studies performed with similar treatment modalities.

## 2. Materials and Methods

### 2.1. Investigational Product

Allocetra-OTS (Enlivex Therapeutics, Ness Ziona, Israel) is manufactured from a mononuclear-enriched cell fraction collected from the peripheral blood of healthy unrelated donors via leukapheresis. Cells and plasma are transported to Enlivex’s manufacturing facility under temperature-controlled conditions. Upon arrival, the cells are processed and cryopreserved according to common practice for the cryopreservation of donor lymphocyte infusion (DLI) cells. Following thawing and the removal of the freezing medium, the cells are resuspended and incubated in apoptosis induction medium containing methylprednisolone, which is subsequently removed using an automated process of media replacement and volume reduction. The apoptotic cells are suspended in Plasma-Lyte, irradiated (X-ray, 4000 cGy), diluted 1:1 with CryoStor^®^ CS5 (BioLife Solutions Inc., Bothell, WA, USA) to a final targeted concentration in a solution containing 2.5% DMSO, and stored in liquid nitrogen. On each dosing day, the vehicle (Allocetra-OTS suspension medium) is thawed in a 37 ± 1 °C water bath for up to 5 min, transferred to a sterile bottle under aseptic conditions, and kept at ambient conditions. Allocetra-OTS cells are thawed (37 ± 1 °C water bath for approx. 2 min), centrifuged (300× *g*, 10 min at 2–8 °C), and resuspended using the vehicle. The final cell concentration is adjusted using the vehicle, according to the required injected dose. Cells are readministered no later than 2 h after thawing.

### 2.2. Animal Husbandry and Maintenance

SD rats were chosen as the animal species since they are commonly used in safety studies in accordance with international recommendations and the published literature. The SD strain is a well-known laboratory model with adequate historical data. A total of 120 SD rats (60 males (M) and 60 females (F)), aged 8 weeks at study initiation, were obtained from Envigo CRS Ltd. (Ness-Ziona, Israel) and were housed and treated at Pharmaseed Ltd. (Ness-Ziona, Israel). Animals were provided with a commercial rodent diet ad libitum and allowed free access to drinking water. Temperature was maintained at 18–24 °C with 30–70% relative humidity and a 12 h light/dark cycle. The study was performed in compliance with the Israel Animal Welfare Act and was approved by the Israel Board for Animal Experiments Ethics Committee (#NPC-Ph-IL-2111-113-5).

### 2.3. Experimental Design

Allocetra-OTS was studied at three doses (140 × 10^6^ [group 2M/2F], 700 × 10^6^ [group 3M/3F] and 1260 × 10^6^ Allocetra-OTS cells/kg [group 4M/4F]) and compared to the vehicle group (in suspension media; group 1M/1F). Allocetra-OTS or vehicle was administered IV (tail vain) via three consecutive administrations (days 1–3). The animals of the main study were sacrificed on day 4, and the animals of the recovery groups (Allocetra-OTS high dose and vehicle) were kept for two or four weeks of recovery after the last dosing day (see Table 1). Allocetra-OTS or the vehicle were administered to 5–10 animals per group at a rate of 2 mL/min at a dose volume of 10 mL/kg (Table 1). The study was conducted in compliance with the principles of Good Laboratory Practice (GLP). The details regarding the design of the experiment, the groups’ allocation across the study phases (main study, 14 days recovery phase, and 28 days recovery phase), and the treatment doses are described in Table 1.

### 2.4. Observations and Examinations

All animals were observed for clinical signs prior to each dose for the first 3 h post first dosing, for 1 h post the second and the third doses, and twice a week thereafter for the recovery phases before termination. Body weight and food consumption were monitored, along with ophthalmic examinations. Prior to termination, urine was collected from the animals of both the main and recovery phases, and analyzed for pH, glucose, specific gravity, bilirubin, urine total protein, ketones, urobilinogen, and microscopic sediment. Upon termination, and following at least 2 h of food restriction, blood was drawn from all animals for the following analyses:

Hematology: A total of 300 µL of whole blood in K3 EDTA-containing tubes was run on an Advia 2120i blood analyzer (automatic differential count) (Siemens HealthCare Ltd., Rosh Haayin, Israel) or 50 µL of whole blood using the Sysmex blood analyzer (manual differential count).

Clinical Chemistry: Approx. 0.5 mL of whole blood per animal was collected into cup tubes with clotting activator gel. The tubes were kept at room temperature for at least 30 min for clotting and then centrifuged at 4 °C for 10 min at 1790× *g*. Separated serum (180 µL) was analyzed with a Cobas 6000 chemistry analyzer.

Coagulation: Citrated blood (approx. 450 µL of blood + 50 µL of sodium citrate or approx. 900 µL of blood + 100 µL of sodium citrate) per animal was collected into sodium citrate tubes. The samples were centrifuged at 4 °C for 10 min at 1790× *g* for the separation of plasma. Citrated plasma (300 µL) was analyzed for prothrombin time (PT), activated partial thromboplastin time (aPTT), and fibrinogen (FIB) using a Sysmex CS2500 analyzer (Sysmex, Singapore). Group mean values were compared to the normal references (strain- and sex-related values) provided by the clinical pathology laboratory and analyzed by *t*-test for statistical differences from the relevant vehicle control group.

Urine Analysis: Prior to termination, urine was collected from both the main and recovery phases during 14 ± 2 h using metabolic cages. The urine samples were analyzed for the following parameters: pH, glucose, specific gravity, bilirubin, urine total protein, ketones, urobilinogen—qualitative determination, and microscopic examination of sediment.

Gross Pathology: Gross pathology was thoroughly examined, and organs were wet weighed. All groups were subjected to histopathology analysis of the following tissues: adrenals, aorta, brain, cecum, colon, ileum with Peyer’s patches, prostate, seminal vesicles and coagulating glands, duodenum, stomach, epididymides, esophagus, thyroid with parathyroids, trachea, eyes, femoral muscle with sciatic nerve, femur with joint, heart, jejunum, kidneys, liver, lung including main bronchi, skin with mammary glands, mandibular lymph nodes, ovaries and oviducts, pituitary, pancreas, rectum, urinary bladder, salivary glands, spinal cord, spleen, sternum with marrow, testes, thymus, uterus with cervix, vagina, mesentery, and tail (injection site).

Histopathological Assessment: Histology slide preparation included tissue decalcification (as applicable), trimming, dehydration, and embedding. The resulting blocks were sectioned to a thickness of approx. 4 microns, mounted, and stained with hematoxylin and eosin. Histopathology changes were described and scored using a semi-quantitative grading (0 = normal, 1 = minimal, 2 = mild, 3 = moderate, 4 = severe) [34].

## 3. Results

### 3.1. Morbidity and Mortality, Clinical Signs, and Body Weights

No animal died or was found in morbid condition during the study, and no significant differences in food consumption were observed between the tested groups. No differences in average body weight were observed between the vehicle- (1F) and the Allocetra-OTS-treated groups (2F, 3F, and 4F) in female rats of the main and recovery phases. However, on days 2 and 3 in male rats in both the main and the 14-day recovery phase studies, a slight (<3%) but statistically significant decrease was observed in the relative average body weight (% of day 1 weight) of group 4M compared to the vehicle group (1M). This decrease was also observed on day 3 in group 3M of the main study. Full recovery of body weight was seen in the 28-day recovery phase.

All animals in both the main and the recovery phases of the study demonstrated piloerection, swelling of the feet and face, edema, and cyanosis during the first 3 h after the initial administration of both the vehicle and Allocetra-OTS. These phenomena were also observed after the second and third administrations. Most animals demonstrated full recovery from all symptoms by 24 h post-administration. These clinical signs had disappeared by the end of the injection days (on day 4) in all cases, both in the main and in the recovery studies. As was seen in the vehicle as well, these clinical signs were not all considered to be Allocetra-OTS-related. Furthermore, no Allocetra-OTS-related ophthalmologic symptoms were observed in any of the animals.

### 3.2. Clinical Pathology

The comprehensive findings from the hematology, clinical chemistry, and coagulation analysis are summarized in tables presented in the Supplementary information (Tables S1–S15) as values of the averages per group. A distinct table is provided for the treatment groups categorized by gender (male or female groups) and for both the main study and recovery phases for each clinical pathology analysis. For each treatment group, *t*-test analyses were used to compare the result to the normal range of each parameter, as well as to the vehicle-treated group.

#### 3.2.1. Hematology

White blood cells (WBCs): Among the male rat groups in the main study, a significant dose-related increase in WBC concentration was observed in all the treated groups (2M, *p* < 0.01; 3M and 4M, *p* < 0.001) compared to the vehicle group (1M), although WBC values remained within the normal range for all groups. This increase was not observed in any of the recovery phases.

Lymphocytes: Among the male rats in the main study, a significant dose-related increase in the absolute lymphocyte count was observed in all the treated groups (2M, *p* < 0.01; 3M and 4M, *p* < 0.001) compared to the vehicle group (1M). A significant increase (*p* < 0.05) was also observed in the female group (3F) of the main study compared to the vehicle group (1F). This increase was not observed in any of the recovery phases. All of the increased values were within their normal ranges.

Monocytes: Among the female rat groups in the main study, both the absolute count and the average percentage of monocyte values were significantly increased in groups 3F (*p* < 0.05) and 4F (*p* < 0.01) compared to the vehicle group (1F). A significant increase in the absolute monocyte count was also observed in the male rat groups (3M, *p* < 0.01 and 4M, *p* < 0.001), whereas an increase in the monocyte percentage values was observed only in group 4M (*p* < 0.05). Both the absolute and percentage values of the monocytes in all groups remained within normal ranges. No such increases in monocyte values were observed in any of the recovery phases.

Basophils: In both the males and females in the treated groups (2M/2F, 3M/3F, and 4M/4F) of the main study, a significant dose-related increase in both the absolute counts and the percentage values of basophils was observed compared to the vehicle group (1M/1F). Moreover, basophil percentage values were higher than the normal range in both the male and female groups of the main study. This increase was not observed in any of the recovery phases.

All of the abovementioned changes in hematology were related to Allocetra-OTS and were already resolved by the end of the 14-day recovery phase. Most remained within their normal ranges and were therefore not considered to be adverse events (see Supplementary Information).

#### 3.2.2. Clinical Chemistry

Albumin and globulin: In both the male and the female groups of the main study, a significant dose-related decrease in the albumin concentration, all within the normal range, was observed in all the treated groups (2M/2F, 3M/3F, and 4M/4F) compared to the vehicle groups (1M/1F). No such decrease was observed in any of the recovery phases. Among female rats in the main study, a significant dose-related increase in the globulin concentration was observed in all the treated groups (2F, 3F, and 4F) compared to the vehicle group (1F). All observed changes remained within the normal range. A similar increase, again within the normal range, was also observed in the male rat group 4M of the main study compared to the vehicle group (1M) and was not observed in any of the recovery phases. Among female rat groups of the main study, the dose-related albumin/globulin ratio was significantly decreased in all the treated groups (2F, 3F, 4F) compared to the vehicle group (1F). This decrease was also observed in the male rat group (4M) of the main study compared to the vehicle group (1M), but not in any of the recovery phases.

LDH: Among the female rat groups within the main study, the LDH concentration was significantly decreased (within normal range) in two groups (2F, *p* < 0.05; 4F, *p* < 0.01) compared to the vehicle group (1F). A similar decrease (within normal range) was also observed in the treated female 14-day recovery group (4F) but not in the 28-day recovery phase.

SGOT and SGPT: Among the female rat groups within the main study, a significant decrease in the serum glutamic-oxaloacetic transaminase (SGOT) concentration (though within its normal ranges) was observed in groups 2F and 3F compared to the vehicle group (1F). This decrease was not observed in any of the recovery phases. In group 3F of the main study, the serum glutamate pyruvate transaminase (SGPT) concentration was also significantly decreased (also within its normal range) compared to the vehicle group (1F), but no such decrease was seen in any of the recovery phases. In addition, in the recovery phase, the SGPT values among treated female rats (4F) were higher than their normal range, but they did not differ statistically from values in the vehicle group.

Calcium, phosphorus, and potassium: In group 3M of the main study, a slight but significant increase (within its normal range) in the calcium concentration was observed compared to the vehicle group (1M). This increase was not observed in any of the recovery phases. In group 4M of the main study, a significant increase (within normal range) in the phosphorus concentration was observed compared to the vehicle group (1M). No such increase was observed in any of the recovery phases. In group 3M of the main study, the potassium concentration was significantly decreased (within normal range) in comparison to the vehicle group (1M). A similar decrease was observed in treated males (4) in the 14-day recovery group (4M).

Creatinine: In the female vehicle group of the main study, creatinine values were slightly lower than their normal range. In group 3F of the main study, the creatinine concentration was slightly (within normal range) but significantly increased compared to the vehicle group (1F). This slight increase was not observed in any of the recovery phases.

Total protein: Among the male rat groups of the main study, a significant dose-related decrease, within its normal range, in the total protein concentration was observed in all the treated groups (2M, 3M, 4M) compared to the vehicle group (1M). This decrease was not observed in any of the recovery phases.

#### 3.2.3. Coagulation

Fibrinogen: Among the male rat groups (3M and 4M) of the main study, the fibrinogen concentration was significantly increased compared to the vehicle group (1M), with full recovery attained by day 14.

aPTT: In the male rat high-dose treatment group (4M), both in the main study and in the 28-day recovery phase, a slight (within normal range) but significant increase in aPTT was observed in comparison to the vehicle group (1M).

In general, some significant differences in clinical chemistry parameters were observed in both male and female rat groups of the main study. No such differences were observed in either the 14-day or the 28-day recovery phases, except for the decrease in the LDH concentration observed in the 14-day (but not in the 28-day) recovery phase in female rats, and the decrease in the potassium concentration that was also observed in the males of the 14-day recovery phase, but not in the 28-day recovery phase. In general, male rats seemed to be more susceptible to the transient recorded effects than females.

### 3.3. Urinalysis

There was no difference in urine parameters between the vehicle- and the drug-treated groups in the main study and the recovery phases.

### 3.4. Organ Weight

A significant dose-related increase (*p* < 0.001) in absolute and relative spleen weights was observed in all the treated groups of the main study compared to the vehicle groups. A significant increase in absolute and in relative spleen weight was also observed in male treated rats (4M) of the 28-day recovery phase (*p* < 0.05). The relative spleen weights were calculated as a percent (%) of the total body weight of each animal. The average of the relative spleen weight results across all the treatment groups during both the main study and the recovery phases is presented in Table 2.

### 3.5. Gross Pathology

The most prominent abnormality was the enlarged spleen observed in most of the treated male and female rat groups of the main study, including those in the vehicle groups. This phenomenon was not observed in the 28-day recovery phase.

### 3.6. Histopathological Evaluation

The histopathological individual findings are presented in the Supplementary Tables as follows: Tables S16–S19, group 1M–4M (male), respectively, main study. Tables S20–S23, group 1F–4F, respectively (female), main study. Table S24, 1M and 4M (male), recovery phase 14 days. Table S25, 1F and 4F (female), recovery phase 14 days. Table S26, 1M and 4M (male), recovery phase 28 days. Table S27, 1F and 4F (female), recovery phase 28 days.

**Figure 2:**
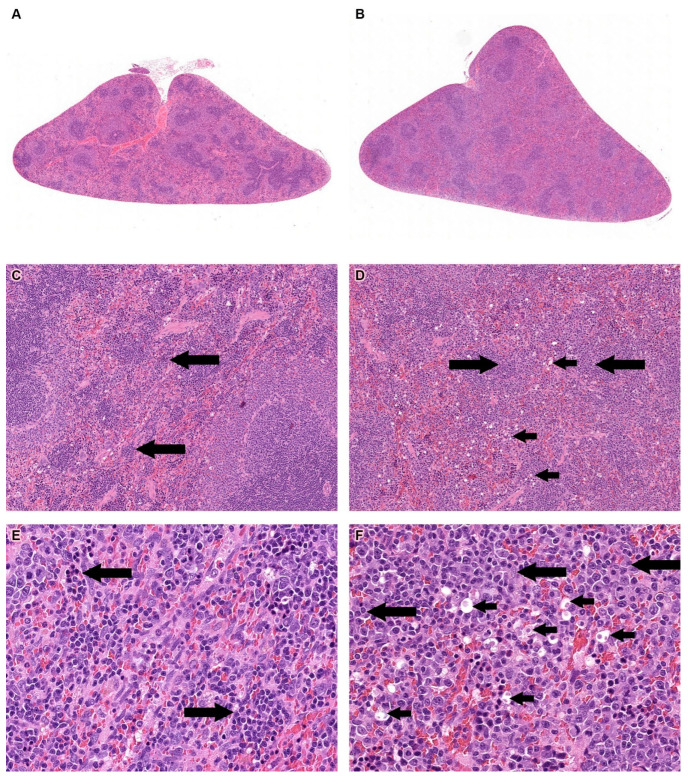
Histopathology analysis of spleen. (**A**). Histopathology section of the spleen from a rat from the vehicle group (1M). Original magnification ×1. (**B**) Spleen from the high-dose Allocetra-OTS-treated animal group (4M). Original magnification ×1. (**C**) Spleen from the vehicle group (1M). Long arrows indicate minimal extramedullary hematopoiesis (EMH) in the red pulp. Original magnification ×10. (**D**) Spleen from the high-dose Allocetra-OTS group (4M). Short arrows indicate the presence of apoptotic cells. Long arrows indicate EMH. Original magnification ×10. (**E**) Spleen from the vehicle group (1M). Long arrows indicate EMH in the red pulp. Original magnification ×10. (**F**) Spleen from the high-dose Allocetra-OTS group (4M). Short arrows indicate the presence of apoptotic cells, and long arrows indicate EMH.

Treatment-related changes were observed only in the red pulp of the spleen. These changes included the presence of round, relatively small hyperchromatic dark blue nuclear remnants, and/or round empty cavities with or without remaining nuclear and cell eosinophilic ghosts (dead cells) (Figure 2A–F and Tables S16–S27). These changes were consistent with a diagnosis of apoptotic cells [35]. The degree of change was mild in groups 3 and 4, and minimal in group 2. In addition, extramedullary hematopoiesis (EMH), manifested as a diffuse increase in the number of cells with a large irregular nucleus, prominent nucleoli, and scant cytoplasm (compared to controls), was observed in the Allocetra-OTS-treated rats. The degree of change was usually moderate in groups 3 and 4 and mild in group 2, and minimal in the vehicle groups (Figure 2A–F and Tables S16–S27). Extramedullary hematopoiesis (EMH) was observed in the red pulp of the spleen, with no adverse events, and was considered to be a normal and expected reaction for the removal of degenerated cells following the IV administration of cell-based therapies [36]. No treatment-related changes were seen in the recovery phases of the study.

## 4. Discussion

Here we present the results of a detailed safety evaluation of three repeated doses of Allocetra-OTS (140 × 10^6^, 700 × 10^6^, and 1260 × 10^6^ cells/kg) administered IV in SD rats and compared to a control vehicle group. The dosages used in this toxicology study were at least nine-fold higher than those of the equivalent human doses administered in a previously conducted phase 1b clinical study of Allocetra-OTS in sepsis [3], as well as in an ongoing sepsis phase 2 study.

All dose levels were well tolerated, and no serious adverse or toxicologically meaningful effects were observed with regard to body weight, clinical signs, food consumption, urinalysis, or ophthalmologic evaluation.

Piloerection, swelling, edema, and cyanosis were observed in all animals during the first 3 h after the dosage administration in both the vehicle- and Allocetra-OTS-treated groups. These changes were fully resolved in all cases. These changes were considered to be vehicle-related, as also confirmed in a follow-up experiment, and were probably caused by the hypersensitivity of rats to dextran, a component of the media formulation. This reaction is well known in rats and is not expected in humans [37,38,39,40].

Some significant differences in the hematology analysis parameters were observed in both the male and female rat groups of the main and recovery phases, found while comparing the treated and vehicle groups. Since most of these differences were not related to dosage, or disappeared during recovery phases, they were not considered to be toxicologically adverse events. The high WBC levels observed in the male groups in the main study are an expected phenomenon since -OTS consists of foreign (human) cells that lead to leukocyte proliferation as part of the physiological immune response [41]. Significant differences were observed in several of the clinical chemistry parameters in both the male and female rat groups of the main study. These differences were not considered to be clinically relevant, as well as the decreases in the LDH concentration and potassium that were not seen in the 28-day recovery phase.

Significant dose-related increases in absolute and relative spleen weights were observed in all the drug-treated groups of the main study compared to vehicle-treated groups, including in the 28-day recovery phase in male rats. These findings correlated with enlarged spleens, with the most prominent abnormality being observed during the evaluation of gross pathology in most of the Allocetra-OTS-treated groups of the main study. Notably, differences in recovery phases between the treated and vehicle groups were substantially smaller and were not observed during the gross pathology evaluation after 28 days of recovery. IV-injected cells have a well-known tendency to become trapped in the spleen. This organ serves as a common homing location for immune cells when infused systemically [42,43,44] and was shown to be the major target for the accumulation of PBMCs following IV injection in rats [45]. This phenomenon is also observed in other cell-based treatments such as stem cell therapies [46,47]. It is, therefore, not surprising to observe the presence of PBMC-derived apoptotic cells in the spleen in this study. Nevertheless, other than the expected splenomegaly, this did not lead to any observed adverse events in the spleen.

This phenomenon was further investigated, moreover, in an in vivo study in mice, aimed at determining whether the observed splenomegaly is restricted to the xenogeneic source of apoptotic cells (namely, human cells injected into BALB/c mouse). That study compared the impact of repeated injections of apoptotic cells using xenogeneic, allogeneic (C57BL/6 cells to BALB/c mice, reflecting the clinical administration of allogeneic Allocetra-OTS to humans), and syngeneic (BALB/c cells to BALB/c mice) apoptotic cells. The results demonstrated splenomegaly in the human Allocetra-OTS-treated mice, but not in the syngeneic- or allogeneic-treated mice. Despite several differences in the immune response between Wistar rats and BALB/c mice to foreign antigens (human cells), the central ‘eat me’ signal pathways for apoptotic cell engulfment from C. elegans to humans are well conserved [48,49]. We therefore believe that the findings of that in vivo mouse study adequately support our splenomegaly finding obtained in this toxicology study.

Since the injection of Allocetra-OTS into humans in a clinical setting is the equivalent of an allogeneic apoptotic cell injection into mice, it can be hypothesized that the changes observed in the spleens of Allocetra-OTS-treated mice are related to their xenogeneic origin and are not expected in an allogeneic clinical setting.

An additional observed finding is EMH, i.e., the presence of hematopoiesis in locations other than the bone marrow [50,51,52], which has been observed in all animals in this study and was more pronounced in the Allocetra-OTS-treated rats. EMH is an additional factor contributing to the splenomegaly and the changes seen in the WBC counts observed here in the Allocetra-OTS-treated animals. Commonly observed in rats [53], EMH is not limited to hematotoxic insults and can also occur in various conditions such as general stress, inflammation, and systemic anemia. In all the animals in our study, including those receiving the highest dose of Allocetra-OTS and experiencing the highest degree of EMH and accumulation of apoptotic cells, the findings were completely resolved during the 14-day recovery phase. Together with the fact that EMH was also observed in the vehicle group, the observed EMH was not regarded as related to Allocetra-OTS treatment. This conclusion is in line with the results from a previous GLP toxicology study in which Allocetra-OTS was administered at the same doses and with the dose regimen applied in the current study to SD rats with a former (liquid) formulation. In that study, the spleens of animals in both the control and high-dose groups (1260 cells × 10^6^/kg) demonstrated the presence of apoptotic cells and EMH, which were both more pronounced in Allocetra-OTS-treated rats. Based on those previous results, it can be concluded that the EMH observed in all animals in the present study indeed reflects the presence of Allocetra-OTS cells in the spleen.

Given that the spleen was the only target organ, and that the changes noted in this organ are considered to reflect the expected pharmacological accumulation of IV-administered Allocetra-OTS cells (associated with reactive EMH, which recovered completely following the 14 days of drug withdrawal), the observed changes are judged to be not adverse according to the criteria of the Society of Toxicologic Pathology [54,55,56,57,58,59].

In general, Allocetra-OTS was well tolerated in our SD rat model, and no serious adverse or toxicologically meaningful effects were observed. It was therefore concluded that Allocetra-OTS is safe, with the highest dose administered determined as No Observed Adverse Effect Level (NOAEL). This safety profile is comparable to a previously conducted GLP toxicology study evaluating the safety of a liquid formulation of Allocetra-OTS in SD rats using identical doses and the same dose regimen used here, in which the highest administered dose was also determined as NOAEL. Since this toxicity preclinical examination revealed no signs of abnormal cell proliferation in any organ, and no threshold for adverse effects on the host was detected [33,60], we believe that the present study confirms the favorable safety profile of Allocetra-OTS as a possible treatment modality for humans in clinical trial settings. It also provides critical information on the expected changes in animal models when injected IV with PBMC-derived apoptotic cells, thus facilitating the interpretation of histopathology findings in future studies of such treatments.

## Figures and Tables

**Table 1:** Experimental design.

	Group	Animal No. (Total No.)		IV Treatment	Dose (Cells × 10^6^/kg)	Dose Volume (mL/kg)
		M	F			
Main Study	1M/1F	10	10	Vehicle	NA	10
	2M/2F	10	10	Allocetra-OTS	140	
	3M/3F	10	10		700	
	4M/4F	10	10		1260	
Recovery Phase—14 days	1M/1F	5	5	Vehicle	NA	10
	4M/4F	5	5	Allocetra-OTS	1260	
Recovery Phase—28 days	1M/1F	5	5	Vehicle	NA	10
	4M/4F	5	5	Allocetra-OTS	1260	

M, male; F, female; NA, not applicable; IV, intravenous.

**Table 2:** Relative spleen weight analysis.

**Relative Organ Weight** **(% of BW)**	**Vehicle (1M/1F)**			**Allocetra-OTS 140 × 10^6^ cells/kg (2M/2F)**			**Allocetra-OTS 700 × 10^6^ cells/kg (3M/3F)**			**Allocetra-OTS 1260 × 10^6^ cells/kg (4M/4F)**		
	**AVG**	**SEM**	**N**	**AVG**	**SEM**	**N**	**AVG**	**SEM**	**N**	**AVG**	**SEM**	**N**
Males, main study												
Spleen	0.263	0.010	10	0.395 ***	0.012	10	0.449 ***	0.013	10	0.456 ***	0.010	10
Females, main study												
Spleen	0.292	0.014	10	0.430 ***	0.017	10	0.468 ***	0.013	10	0.489 ***	0.017	10
**Relative Organ Weight** **(% of BW)**	**Vehicle (1M/1F)**					**Allocetra-OTS 1260 × 10^6^ cells/kg (4M/4F)**						
	**AVG**	**SEM**			**N**	**AVG**		**SEM**			**N**	
Males, recovery, 14 days												
Spleen	0.260	0.013			5	0.294		0.008			5	
Females, recovery, 14 days												
Spleen	0.296	0.017			5	0.332		0.005			5	
Males, recovery, 28 days												
Spleen	0.223	0.004			5	0.265 *		0.013			5	
Females, recovery, 28 days												
Spleen	0.265	0.012			5	0.302		0.012			5	

* *p* < 0.05; *** *p* < 0.001 compared to vehicle (1M) using *t*-test. BW = body weight; F = female; M = male; N = number of animals; AVG = average; SEM = standard error of the mean.

## Data Availability

The raw data supporting the conclusions of this article will be made available by the authors on request.

## Supplementary Materials

The following supporting information can be downloaded at: https://www.mdpi.com/article/10.3390/pharmaceutics16030426/s1, Figure S1: Average Allocetra-OTS concentration vs. time data following IV administration, linear scale; Table S1: Hematology analysis, Male, Main Study; Table S2: Hematology analysis, Female, Main Study; Table S3: Hematology analysis, Male, Recovery Phase 14 Days; Table S4: Hematology analysis, Female, Recovery Phase 14 Days; Table S5: Hematology analysis, Male, Recovery Phase 28 Days; Table S6: Hematology analysis, Female, Recovery Phase 28 Days; Table S7: Clinical chemistry, Male, Main Study; Table S8: Clinical chemistry, Female, Main Study; Table S9: Clinical chemistry, Male, Recovery 14 Days Phase; Table S10: Clinical chemistry, Female, Recovery 14 Days Phase; Table S11: Clinical chemistry, Male, Recovery 28 Days Phase; Table S12: Clinical chemistry, Female, Recovery 28 Days Phase; Table S13: Coagulation, Males and Females, Main study; Table S14: Coagulation, Males and Females, Recovery 14 Days Phase; Table S15: Coagulation, Males and Females, Recovery 28 Days Phase; Table S16: Individual histopathological findings-Male Main Study (Group 1M); Table S17: Individual histopathological findings-Male Main study (Group 2M); Table S18: Individual histopathological findings-Male Main study (Group 3M); Table S19: Individual histopathological findings-Male Main Study (Group 4M); Table S20: Individual histopathological findings-Female Main Study (Group 1F); Table S21: Individual histopathological findings-Female Main study (Group 2F); Table S22: Individual histopathological findings-Female Main study (Group 3F); Table S23: Individual histopathological findings-Female Main Study (Group 4F); Table S24: Individual histopathological findings-14 days Recovery Phase, Groups 1M and 4M; Table S25: Individual histopathological findings-14 days Recovery Phase, Groups 1F and 4F; Table S26: Individual histopathological findings-28 days Recovery phase, Group 1M and 4M; Table S27: Individual histopathological findings-28 days Recovery phase, Group 1F and 4F.
