# Supplementary material for: Safety of Repeated Administration of Xenogeneic Human Apoptotic State (Allocetra-OTS) in Sprague Dawley Rats
Source: Pharmaceutics. 2024 Mar 20;16(3):426. doi: 10.3390/pharmaceutics16030426 (PMC10975356; doi:10.3390/pharmaceutics16030426)
Supplement: Supplementary file 1 [file pharmaceutics-16-00426-s001.zip › pharmaceutics-2915323-supplementary.pdf]

Supplementary Figures and Tables

Safety of repeated administration of xenogeneic human apoptotic state (Allocetra-OTS) in Sprague Dawley rats

Figure S1: Average Allocetra-OTS concentration vs. time data following IV administration, linear scale

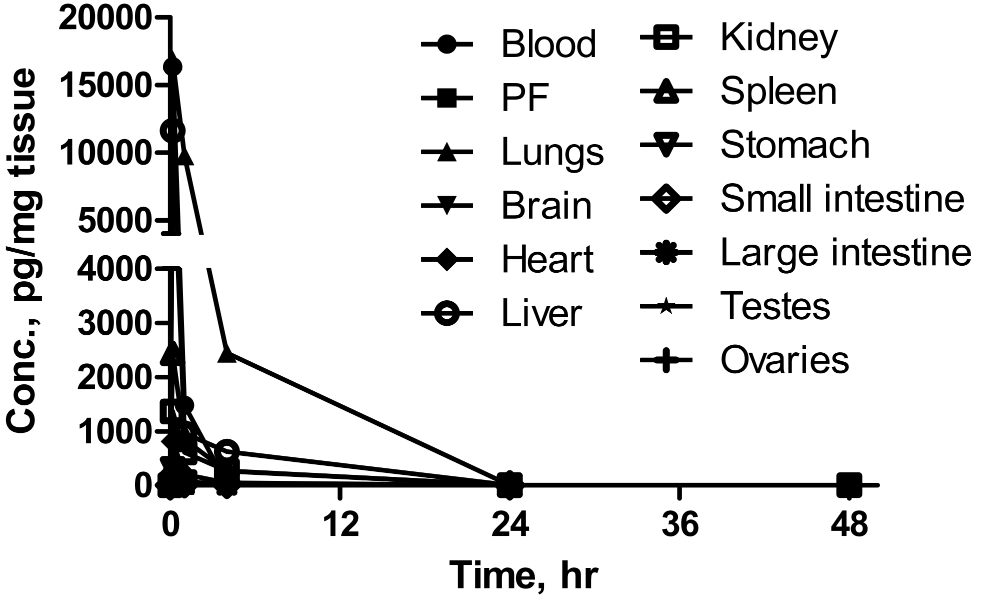

FIGURE LEGENDS

Figure S1. Average Allocetra-OTS concentration vs. time data following IV administration, linear scale. Biodistribution GLP-study results following single IV administration of Allocetra-OTS to ICR mice. The concentration of Allocetra-OTS in different mouse organs, tissues and body fluids was quantified using a highly sensitive quantitative polymerase chain reaction (qPCR) method based on the quantification of human unique Alu sequences. The analysis results are reported as the relative human to mouse DNA concentration

Table S1: Hematology analysis, Male, Main Study

Group	Normal Range	Vehicle (1M)			Allocetra-OTS 140x10^6 cells/kg (2M)			Allocetra-OTS 700x10^6 cells/kg (3M)			Allocetra-OTS 1260x10^6 cells/kg (4M)		
		AVG	SEM	N	AVG	SEM	N	AVG	SEM	N	AVG	SEM	N
WBC (10*3/µL)	**6.4-18.8**	6.48	0.32	10	8.06**	0.42	10	10.14***	0.66	10	11.63***	0.54	10
RBC (10*6/µL)	**7.8-9.38**	**7.40**	0.11	10	**7.28**	0.13	10	**7.17**	0.08	10	**7.31**	0.06	10
HGB (g/dL)	**14.6-16.8**	**14.26**	0.15	10	**14.37**	0.21	10	**14.15**	0.13	10	**14.47**	0.11	10
Hematocrit (%)	**45.4-54.1**	46.78	0.58	10	46.97	0.81	10	45.75	0.42	10	47.04	0.53	10
MCV (fL)	**53.3-62.4**	**63.24**	0.61	10	**64.56**	0.61	10	**63.83**	0.71	10	**64.33**	0.55	10
MCH (pg)	**17-19.6**	19.29	0.23	10	**19.78**	0.18	10	**19.74**	0.19	10	**19.80**	0.16	10
MCHC (g/dL)	**29.6-33.6**	30.53	0.18	10	30.63	0.18	10	30.93	0.18	10	30.80	0.22	10
Neutrophils (%)	**5.0-31.0**	18.85	1.90	10	13.93*	1.25	10	13.31*	0.71	10	20.85	2.67	10
Bands (%)	**NA**	0.00	0.00	10	0.00	0.00	10	0.00	0.00	10	0.00	0.00	10
Lymphocytes (%)	**59-93**	76.63	1.89	10	80.26	1.48	10	80.11	1.16	10	73.61	2.73	10
Monocytes (%)	**0-6**	2.00	0.13	10	1.87	0.15	10	2.31	0.22	10	2.49*	0.12	10
Eosinophils (%)	**0-4**	1.40	0.19	10	2.01	0.38	10	2.30	0.69	10	1.08	0.11	10
Basophils (%)	**0-2**	0.12	0.01	10	**0.29*** **	0.03	10	**0.31*** **	0.02	10	**0.30*** **	0.03	10
Platelets (10*3/µL)	**453-1081**	722.20	67.15	10	835.70	24.34	10	795.30	51.60	10	762.00	63.97	10
Retic (%)	**NA**	3.556	0.18	10	2.98*	0.17	10	3.15	0.17	10	2.28**	0.30	10
MPV (FI)	**NA**	9.64	0.38	10	8.93	0.20	10	9.46	0.22	10	9.96	0.65	10
RDW (%)	**NA**	12.01	0.14	10	11.57*	0.11	10	11.59*	0.11	10	12.52	1.19	10
Neutrophils Absolute (10*3/µL)	**NA**	1.23	0.15	10	1.13	0.13	10	1.33	0.08	10	2.44**	0.34	10
Bands Absolute (10*3/µL)	**NA**	0.00	0.00	10	0.00	0.00	10	0.00	0.00	10	0.00	0.00	10
Lymphocyte Absolute (10*3/µL)	**NA**	4.96	0.26	10	6.46**	0.34	10	8.16***	0.59	10	8.55***	0.51	10
Monocyte Absolute (10*3/µL)	**NA**	0.13	0.01	10	0.15	0.02	10	0.24**	0.03	10	0.29***	0.01	10
Eosinophils Absolute (10*3/µL)	**NA**	0.09	0.01	10	0.16	0.03	10	0.21*	0.04	10	0.13	0.01	10
Basophils Absolute (10*3/µL)	**NA**	0.01	0.00	10	0.02***	0.00	10	0.03***	0.00	10	0.04***	0.00	10

M= Male; AVG= Average; SEM= Standard Error of the Mean; N= Number; *p<0.05, **p<0.01, ***p<0.001 compared to Vehicle (1M) using T-test

Table S2: Hematology analysis, Female, Main Study

Group	Normal Range	Vehicle (1F)			Allocetra-OTS 140x10^6 cells/kg (2F)			Allocetra-OTS 700 x10^6 cells/kg (3F)			Allocetra-OTS 1260 x10^6 cells/kg (4F)		
		AVG	SEM	N	AVG	SEM	N	AVG	SEM	N	AVG	SEM	N
WBC (10*3/µL)	5.3-16.7	6.35	0.36	10	6.81	0.21	10	7.43	0.50	10	7.02	0.49	10
RBC (10*6/µL)	7.4-9.28	**7.38**	0.05	10	**7.37**	0.10	10	**7.32**	0.07	10	**7.32**	0.08	10
HGB (g/dL)	14-16.9	14.56	0.14	10	14.55	0.21	10	14.39	0.14	10	14.52	0.12	10
Hematocrit (%)	42.7-52.4	45.58	0.45	10	45.68	0.62	10	45.36	0.49	10	45.82	0.45	10
MCV (fL)	53.1-61.0	**61.82**	0.55	10	**62.01**	0.51	10	**62.05**	0.90	10	**62.68**	0.75	10
MCH (pg)	17.2-19.8	19.77	0.17	10	19.74	0.16	10	19.67	0.20	10	**19.87**	0.13	10
MCHC (g/dL)	31.0-34.0	32.00	0.28	10	31.88	0.22	10	31.74	0.20	10	31.74	0.27	10
Neutrophils (%)	5.0-19.0	**23.10**	2.34	10	17.72	2.70	10	15.00*	1.57	10	**19.34**	2.12	10
Bands (%)	NA	0.00	0.00	10	0.00	0.00	10	0.00	0.00	10	0.00	0.00	10
Lymphocytes (%)	75-90	**71.54**	2.32	10	76.95	2.81	10	78.30*	1.53	10	**72.50**	2.27	10
Monocytes (%)	0-5	1.32	0.17	10	1.53	0.10	10	1.88*	0.17	10	1.92**	0.12	10
Eosinophils (%)	0-5	3.01	0.47	10	2.03	0.28	10	2.65	0.31	10	3.87	0.77	10
Basophils (%)	0-2	0.14	0.02	10	**0.27****	0.03	10	**0.28****	0.03	10	**0.32*****	0.02	10
Platelets (10*3/µL)	550-1140	624.70	42.34	10	646.10	53.73	10	701.90	18.90	10	583.90	40.10	10
Retic (%)	NA	2.17	0.17	10	2.19	0.08	10	2.44	0.25	10	2.39	0.17	10
MPV (FI)	NA	8.84	0.41	10	8.81	0.33	10	8.95	0.28	10	9.61	0.31	10
RDW (%)	NA	10.75	0.22	10	10.7	0.12	10	10.85	0.09	10	10.71	0.08	10
Neutrophils Absolute (10*3/µL)	NA	1.486	0.18	10	1.215	0.19	10	1.117	0.14	10	1.382	0.19	10
Bands Absolute (10*3/µL)	NA	0	0.00	10	0	0.00	10	0	0.00	10	0	0.00	10
Lymphocyte Absolute (10*3/µL)	NA	4.525	0.26	10	5.234	0.22	10	5.818*	0.42	10	5.082	0.36	10
Monocyte Absolute (10*3/µL)	NA	0.084	0.01	10	0.105	0.01	10	0.141*	0.02	10	0.131**	0.01	10
Eosinophils Absolute (10*3/µL)	NA	0.193	0.03	10	0.141	0.02	10	0.191	0.02	10	0.253	0.03	10
Basophils Absolute (10*3/µL)	NA	0.009	0.00	10	0.017*	0.00	10	0.021**	0.00	10	0.022**	0.00	10

F= Female; AVG= Average; SEM= Standard Error of the Mean; N= Number; *p<0.05, **p<0.01, ***p<0.001 compared to Vehicle (1F) using T-test

Table S3: Hematology analysis, Male, Recovery Phase 14 Days

Group	Normal Range	Vehicle (1M)			Allocetra-OTS 1260x10^6 cells/kg (4M)		
		AVG	SEM	N	AVG	SEM	N
WBC (10*3/µL)	**6.4-18.8**	7.35	1.26	5	6.47	0.88	5
RBC (10*6/µL)	**7.8-9.38**	8.38	0.13	5	7.98	0.22	5
HGB (g/dL)	**14.6-16.8**	15.14	0.28	5	15.22	0.25	5
Hematocrit (%)	**45.4-54.1**	49.84	1.30	5	49.08	0.87	5
MCV (fL)	**53.3-62.4**	59.44	1.04	5	61.58	1.16	5
MCH (pg)	**17-19.6**	18.10	0.37	5	19.12	0.41	5
MCHC (g/dL)	**29.6-33.6**	30.42	0.40	5	31.02	0.13	5
Neutrophils (%)	**5.0-31.0**	18.40	1.95	5	18.60	1.94	5
Bands (%)	**NA**	0.00	0.00	5	0.00	0.00	5
Lymphocytes (%)	**59-93**	76.72	2.16	5	76.36	2.12	5
Monocytes (%)	**0-6**	2.32	0.09	5	1.92	0.16	5
Eosinophils (%)	**0-4**	1.16	0.20	5	1.90	0.34	5
Basophils (%)	**0-2**	0.14	0.05	5	0.18	0.04	5
Platelets (10*3/µL)	**453-1081**	541.60	66.86	5	465.80	59.34	5
Retic (%)	**NA**	2.17	0.39	5	2.06	0.12	5
MPV (Fl)	**NA**	9.76	0.93	5	9.86	0.42	5
RDW (%)	**NA**	11.60	0.31	5	11.16	0.21	5
Neutrophils Absolute (10*3/µL)	**NA**	1.35	0.26	5	1.14	0.06	5
Bands Absolute (10*3/µL)	**NA**	0.00	0.00	5	0.00	0.00	5
Lymphocyte Absolute (10*3/µL)	**NA**	5.65	1.00	5	5.01	0.83	5
Monocyte Absolute (10*3/µL)	**NA**	0.17	0.03	5	0.13	0.02	5
Eosinophils Absolute (10*3/µL)	**NA**	0.08	0.01	5	0.12	0.02	5
Basophils Absolute (10*3/µL)	**NA**	0.01	0.00	5	0.01	0.00	5

M= Male; AVG= Average; SEM= Standard Error of the Mean; N= Number; *p<0.05, **p<0.01, ***p<0.001 compared to Vehicle (1M) using T-test

Table S4: Hematology analysis, Female, Recovery Phase 14 Days

Group	Normal Range	Vehicle (1F)			Allocetra-OTS 1260x10^6 cells/kg (4F)		
		AVG	SEM	N	AVG	SEM	N
WBC (10*3/µL)	**5.3-16.7**	6.41	0.33	5	6.16	1.01	5
RBC (10*6/µL)	**7.4-9.28**	7.76	0.13	5	8.36**	0.03	5
HGB (g/dL)	**14-16.9**	15.06	0.28	5	15.70	0.16	5
Hematocrit (%)	**42.7-52.4**	47.22	0.77	5	49.86*	0.53	5
MCV (fL)	**53.1-61.0**	60.90	0.34	5	59.64	0.54	5
MCH (pg)	**17.2-19.8**	19.42	0.17	5	18.76*	0.14	5
MCHC (g/dL)	**31.0-34.0**	31.90	0.20	5	31.48	0.07	5
Neutrophils (%)	**5.0-19.0**	**22.72**	4.27	5	**25.20**	5.44	5
Bands (%)	**NA**	0.00	0.00	5	0.00	0.00	5
Lymphocytes (%)	**75-90**	**72.18**	4.15	5	**68.84**	5.62	5

Group	Normal Range	Vehicle (1F)			Allocetra-OTS 1260x10^6 cells/kg (4F)		
		AVG	SEM	N	AVG	SEM	N
Monocytes (%)	**0-5**	1.72	0.29	5	2.26	0.28	5
Eosinophils (%)	**0-5**	2.36	0.12	5	2.60	0.54	5
Basophils (%)	**0-2**	0.16	0.02	5	**0.22**	0.04	5
Platelets (10*3/µL)	**550-1140**	610.80	69.28	5	**537.60**	93.67	5
Retic (%)	**NA**	1.67	0.19	5	1.56	0.14	5
MPV (FI)	**NA**	9.24	0.25	5	10.14	1.04	5
RDW %	**NA**	10.42	0.07	5	10.56	0.26	5
Neutrophils Absolute (10*3/µL)	**NA**	1.45	0.29	5	1.76	0.68	5
Bands Absolute (10*3/µL)	**NA**	0.00	0.00	5	0.00	0.00	5
Lymphocyte Absolute (10*3/µL)	**NA**	4.63	0.35	5	4.02	0.27	5
Monocyte Absolute (10*3/µL)	**NA**	0.11	0.02	5	0.15	0.05	5
Eosinophils Absolute (10*3/µL)	**NA**	0.15	0.01	5	0.16	0.04	5
Basophils Absolute (10*3/µL)	**NA**	0.01	0.00	5	0.01	0.01	5

F= Female; AVG= Average; SEM= Standard Error of the Mean; N= Number; *p<0.05, **p<0.01, ***p<0.001 compared to Vehicle (F) using T-test

Table S5: Hematology analysis, Male, Recovery Phase 28 Days

Group	Normal Range	Vehicle (1M)			Allocetra-OTS 1260x10^6 cells/kg (4M)		
		AVG	SEM	N	AVG	SEM	N
WBC (10*3/µL)	**6.4-18.8**	7.76	0.76	5	7.87	0.25	5
RBC (10*6/µL)	**7.8-9.38**	8.35	0.06	5	8.61	0.21	5
HGB (g/dL)	**14.6-16.8**	15.50	0.04	5	15.60	0.19	5
Hematocrit (%)	**45.4-54.1**	48.78	0.46	5	49.08	0.69	5
MCV (fL)	**53.3-62.4**	58.40	0.35	5	57.12	0.97	5
MCH (pg)	**17-19.6**	18.68	0.10	5	18.16	0.39	5
MCHC (g/dL)	**29.6-33.6**	32.00	0.14	5	31.78	0.21	5
Neutrophils (%)	**5.0-31.0**	21.16	5.75	5	24.16	4.70	5
Bands (%)	**NA**	0.00	0.00	5	0.00	0.00	5
Lymphocytes (%)	**59-93**	73.24	5.63	5	69.92	4.80	5
Monocytes (%)	**0-6**	2.40	0.22	5	2.74	0.32	5
Eosinophils (%)	**0-4**	1.96	0.24	5	1.96	0.61	5
Basophils (%)	**0-2**	**0.24**	0.02	5	0.20	0.03	5
Platelets (10*3/µL)	**453-1081**	569.50	46.33	5	520.00	107.52	5
Retic (%)	**NA**	2.04	0.06	5	2.10	0.04	5
MPV (FI)	**NA**	7.88	0.40	5	9.40	1.57	5
RDW %	**NA**	10.82	0.08	5	11.10	0.26	5
Neutrophils Absolute (10*3/µL)	**NA**	1.78	0.65	5	1.93	0.42	5
Bands Absolute (10*3/µL)	**NA**	0.00	0.00	5	0.00	0.00	5
Lymphocyte Absolute (10*3/µL)	**NA**	5.55	0.42	5	5.48	0.34	5
Monocyte Absolute (10*3/µL)	**NA**	0.18	0.02	5	0.22	0.03	5
Eosinophils Absolute (10*3/µL)	**NA**	0.16	0.03	5	0.15	0.04	5
Basophils Absolute (10*3/µL)	**NA**	0.02	0.00	5	0.02	0.00	5

M= Male; AVG= Average; SEM= Standard Error of the Mean; N= Number; *p<0.05, **p<0.01, ***p<0.001 compared to Vehicle (1M) using T-test

Table S6: Hematology analysis, Female, Recovery Phase 28 Days

Group	Normal Range	Vehicle (1F)			Allocetra-OTS 1260x10^6 cells/kg (4F)		
		AVG	SEM	N	AVG	SEM	N
WBC (10*3/µL)	**5.3-16.7**	5.58	0.44	5	5.56	0.48	5
RBC (10*6/µL)	**7.4-9.28**	8.25	0.23	5	7.99	0.09	5
HGB (g/dL)	**14-16.9**	15.30	0.23	5	15.10	0.20	5
Hematocrit (%)	**42.7-52.4**	47.26	1.00	5	46.16	0.70	5
MCV (fL)	**53.1-61.0**	57.34	0.52	5	57.70	0.31	5
MCH (pg)	**17.2-19.8**	18.58	0.26	5	18.86	0.07	5
MCHC (g/dL)	**31.0-34.0**	32.42	0.22	5	32.68	0.14	5
Neutrophils (%)	**5.0-19.0**	17.40	3.28	5	**25.78**	4.82	5
Bands (%)	**NA**	0.00	0.00	5	0.00	0.00	5
Lymphocytes (%)	**75-90**	77.70	3.23	5	**69.12**	4.81	5
Monocytes (%)	**0-5**	2.06	0.28	5	1.60	0.18	5
Eosinophils (%)	**0-5**	1.88	0.22	5	2.66	0.85	5
Basophils (%)	**0-2**	0.16	0.02	5	0.16	0.02	5
Platelets (10*3/µL)	**550-1140**	717.80	55.14	5	611.60	35.98	5
Retic (%)	**NA**	1.64	0.16	5	1.63	0.16	5
MPV (FI)	**NA**	7.66	0.06	5	8.38	0.43	5
RDW %	**NA**	10.38	0.13	5	10.72	0.14	5
Neutrophils Absolute (10*3/µL)	**NA**	0.92	0.11	5	1.45	0.33	5
Bands Absolute (10*3/µL)	**NA**	0.00	0.00	5	0.00	0.00	5
Lymphocyte Absolute (10*3/µL)	**NA**	4.39	0.51	5	3.82	0.35	5
Monocyte Absolute (10*3/µL)	**NA**	0.11	0.02	5	0.09	0.02	5
Eosinophils Absolute (10*3/µL)	**NA**	0.10	0.01	5	0.15	0.05	5
Basophils Absolute (10*3/µL)	**NA**	0.01	0.00	5	0.01	0.00	5

F= Female; AVG= Average; SEM= Standard Error of the Mean; N= Number; *p<0.05, **p<0.01, ***p<0.001 compared to Vehicle (1F) using T-test

Table S7: Clinical chemistry, Male, Main Study

Group	Normal Range	Vehicle (1M)			Allocetra-OTS 140x10^6 cells/kg (2M)			Allocetra-OTS 700x10^6 cells/kg (3M)			Allocetra-OTS 1260x10^6 cells/kg (4M)		
		AVG	SEM	N	Average	SEM	N	AVG	SEM	N	AVG	SEM	N
Creatinine (mg/dL)	**0.27-0.65**	**0.26**	0.01	10	**0.26**	0.01	10	0.28	0.02	10	0.27	0.01	10
Calcium (mg/dL)	**9.92-12.28**	11.97	0.06	10	11.80	0.11	10	11.76*	0.06	10	11.83	0.08	10
Phosphorus(mg/dL)	**8.1-12.1**	9.36	0.17	10	9.39	0.17	10	9.55	0.19	10	9.94*	0.14	10
Glucose (mg/dL)	**50-140**	135.60	2.20	10	**141.00**	2.78	10	133.80	1.08	10	135.10	2.67	10
Urea (mg/dL)	**29.3-59.2**	35.04	1.00	10	32.55	0.73	10	30.56**	0.98	10	32.42	0.99	10
Cholesterol (mg/dL)	**79-137**	**152.60**	3.71	10	**138.00***	4.74	10	**141.30***	3.43	10	135.70**	4.13	10
Total Protein (g/dL)	**5.92-7.46**	6.53	0.05	10	6.36*	0.04	10	6.40*	0.03	10	6.33*	0.05	10
Albumin (g/dL)	**3.96-4.73**	4.72	0.04	10	4.62	0.05	10	4.58*	0.04	10	4.35***	0.06	10
Globulin (g/dL)	**1.69-3.01**	1.81	0.05	10	1.74	0.04	10	1.82	0.05	10	1.98*	0.04	10
Alb/Glob (Ratio)	**-**	2.63	0.09	10	2.67	0.09	10	2.55	0.09	10	2.21**	0.07	10
Total Bilirubin (mg/dL)	**0.03-0.18**	0.05	0.00	10	0.04	0.00	10	0.05	0.00	10	0.05	0.01	10
Alkaline Phosphatase (IU/L)	**81-197**	**247.60**	10.20	10	**222.70**	8.02	10	**230.20**	6.83	10	**236.40**	5.07	10
LDH (IU/L)	**0-2990**	443.30	41.82	10	451.90	46.54	10	517.10	44.33	10	416.10	33.99	10
SGOT (IU/L)	**57-210**	97.30	3.62	10	94.30	1.87	10	103.00	7.26	10	88.50	2.35	10
SGPT (IU/L)	**30-106**	59.50	2.80	10	57.40	1.09	10	60.30	3.11	10	55.30	1.19	10
Triglycerides (mg/dL)	**21-86**	**93.30**	8.19	10	84.60	8.50	10	**87.10**	8.35	10	80.50	3.80	10
CPK (IU/L)	**0-2296**	589.10	75.47	10	656.10	80.06	10	656.40	55.39	10	690.00	100.13	10
Na (mmol/L)	**142-147**	142.00	0.26	10	142.10	0.38	10	142.00	0.26	10	142.10	0.31	10
K (mmol/L)	**5.3-7.3**	6.05	0.09	10	5.89	0.06	10	5.77*	0.08	10	5.89	0.07	10
Chloride (mmol/L)	**94-101**	99.10	0.38	10	99.20	0.42	10	99.40	0.45	10	99.20	0.33	10
GGTP (IU/L)	**0-1**	0.00	0.00	10	0.00	0.00	10	0.00	0.00	10	0.00	0.00	10

M= Male; AVG= Average; SEM= Standard Error of the Mean; N= Number; *$p<0.05$, **$p<0.01$, ***$p<0.001$ compared to Vehicle (1M) using T-test

Table S8: Clinical chemistry, Female, Main Study

Group	Normal Range	Vehicle (1F)			Allocetra-OTS 140x10^6 cells/kg (2F)			Allocetra-OTS 700x10^6 cells/kg (3F)			Allocetra-OTS 1260x10^6 cells/kg (4F)		
		AVG	SEM	N	AVG	SEM	N	AVG	SEM	N	AVG	SEM	N
Creatinine (mg/dL)	0.28-0.65	**0.27**	0.01	10	0.28	0.01	10	0.29*	0.01	10	0.30	0.01	10
Calcium (mg/dL)	10.16-12.03	11.72	0.09	10	11.97	0.10	10	11.72	0.09	10	11.80	0.11	10
Phosphorus(mg/dL)	7.1-11.6	9.25	0.26	10	9.20	0.30	10	8.79	0.11	10	9.52	0.28	10
Glucose (mg/dL)	52-132	127.90	2.24	10	**136.20**	3.60	10	**139.56****	1.78	10	**136.10***	2.44	10
Urea (mg/dL)	28.8-61.3	33.19	1.16	10	34.03	1.26	10	31.07	1.71	10	30.89	1.20	10
Cholesterol (mg/dL)	71-148	130.10	4.66	10	133.50	6.14	10	130.90	4.36	10	133.30	5.65	10
Total Protein (g/dL)	6-7.31	6.44	0.05	10	6.36	0.08	10	6.41	0.08	10	6.52	0.06	10
Albumin (g/dL)	4.20-4.99	4.84	0.06	10	4.74	0.05	10	4.65*	0.06	10	4.61**	0.05	10
Globulin (g/dL)	1.59-2.54	1.60	0.05	10	1.62	0.06	10	1.76*	0.05	10	1.91***	0.04	10
Alb/Glob (Ratio)	-	3.06	0.12	10	2.97	0.13	10	2.66*	0.09	10	2.43***	0.06	10
Total Bilirubin (mg/dL)	0.04-0.21	**0.03**	0.00	10	**0.03**	0.01	10	0.04	0.00	10	**0.03**	0.01	10
Alkaline Phosphatase (IU/L)	50-153	**185.30**	8.30	10	**192.00**	11.33	10	**176.60**	7.69	10	**174.00**	8.50	10
LDH (IU/L)	0-3062	727.60	60.05	10	524.80*	56.97	10	574.20	52.68	10	481.80**	30.67	10
SGOT (IU/L)	70-178	109.60	3.34	10	98.20*	2.79	10	98.70*	3.83	10	102.40	5.12	10
SGPT (IU/L)	30-82	56.30	2.81	10	55.80	2.57	10	48.60*	1.75	10	51.00	1.62	10
Triglycerides (mg/dL)	16-77	73.00	4.53	10	64.30	2.88	10	65.70	5.33	10	64.40	4.82	10
CPK (IU/L)	0-1595	1094.00	246.38	10	619.50	73.75	10	696.30	82.55	10	891.50	294.61	10
Na (mmol/L)	141-148	**139.30**	0.37	10	**139.60**	0.40	10	**140.80****	0.13	10	**139.90**	0.46	10
K (mmol/L)	5.1-6.8	5.68	0.08	10	5.63	0.06	10	5.78	0.06	10	5.72	0.07	10
Chloride (mmol/L)	94-104	99.30	0.40	10	99.20	0.47	10	99.70	0.40	10	99.60	0.37	10
GGTP (IU/L)	0-1	0.00	0.00	10	0.00	0.00	10	0.00	0.00	10	0.00	0.00	10

F= Female; AVG= Average; SEM= Standard Error of the Mean; N= Number; *p<0.05, **p<0.01, ***p<0.001 compared to Vehicle (1F) using T-test

Table S9: Clinical chemistry, Male, Recovery 14 Days Phase

Group	Normal range	Vehicle (1M)			Allocetra-OTS 1260x10^{6} cells/kg (4M)		
		AVG	SEM	N	AVG	SEM	N
**Creatinine (mg/dL)**	**0.27-0.65**	0.31	0.02	5	0.31	0.01	5
**Calcium (mg/dL)**	**9.92-12.28**	11.89	0.06	5	11.92	0.10	5
**Phosphorus(mg/dL)**	**8.1-12.1**	8.90	0.20	5	9.10	0.16	5
**Glucose (mg/dL)**	**50-140**	**140.40**	3.53	5	**142.80**	2.35	5
**Urea (mg/dL)**	**29.3-59.2**	35.10	1.11	5	35.92	1.66	5
**Cholesterol (mg/dL)**	**79-137**	**141.00**	4.86	5	135.40	8.12	5
**Total Protein (g/dL)**	**5.92-7.46**	6.90	0.04	5	6.85	0.19	5
**Albumin (g/dL)**	**3.96-4.73**	4.70	0.11	5	4.56	0.10	5
**Globulin (g/dL)**	**1.69-3.01**	2.20	0.09	5	2.29	0.13	5
**Alb/Glob (Ratio)**	**-**	2.16	0.13	5	2.01	0.12	5
**Total Bilirubin (mg/dL)**	**0.03-0.18**	0.04	0.00	5	0.03	0.00	5
**Alkaline Phosphatase (IU/L)**	**81-197**	**262.60**	13.95	5	**231.20**	8.47	5
**LDH (IU/L)**	**0-2990**	564.60	102.31	5	442.20	58.71	5
**SGOT (IU/L)**	**57-210**	106.80	7.52	5	98.60	2.40	5
**SGPT (IU/L)**	**30-106**	66.80	3.79	5	69.20	2.65	5
**Triglycerides (mg/dL)**	**21-86**	**94.00**	6.19	5	**110.40**	8.87	5
**CPK (IU/L)**	**0-2296**	635.60	67.43	5	682.40	157.40	5
**Na (mmol/L)**	**142-147**	142.00	0.55	5	142.80	0.58	5
**K (mmol/L)**	**5.3-7.3**	5.88	0.12	5	5.54*	0.07	5
**Chloride (mmol/L)**	**94-101**	100.60	0.60	5	**101.20**	0.58	5
**GGTP (IU/L)**	**0-1**	0.00	0.00	5	0.00	0.00	5

M= Male; AVG= Average; SEM= Standard Error of the Mean; N= Number; *p<0.05, **p<0.01, ***p<0.001 compared to Vehicle (1M) using T-test

Table S10: Clinical chemistry, Female, Recovery 14 Days Phase

Group	Normal Range	Vehicle (1F)			Allocetra-OTS 1260x10^6 cells/kg (4F)		
		AVG	SEM	N	AVG	SEM	N
**Creatinine (mg/dL)**	**0.28-0.65**	0.33	0.01	5	0.33	0.03	5
**Calcium (mg/dL)**	**10.16-12.03**	11.40	0.19	5	11.68	0.16	5
**Phosphorus(mg/dL)**	**7.1-11.6**	8.68	0.16	5	8.22	0.49	5
**Glucose (mg/dL)**	**52-132**	**138.80**	3.84	5	**152.20**	9.14	5
**Urea (mg/dL)**	**28.8-61.3**	37.94	2.35	5	37.40	1.40	5
**Cholesterol (mg/dL)**	**71-148**	129.80	7.00	5	142.40	12.15	5
**Total Protein (g/dL)**	**6-7.31**	6.72	0.10	5	6.91	0.09	5
**Albumin (g/dL)**	**4.20-4.99**	4.78	0.10	5	4.98	0.07	5
**Globulin (g/dL)**	**1.59-2.54**	1.94	0.07	5	1.93	0.10	5
**Alb/Glob (Ratio)**	**-**	2.47	0.11	5	2.61	0.16	5
**Total Bilirubin (mg/dL)**	**0.04-0.21**	0.04	0.01	5	0.04	0.01	5
**Alkaline Phosphatase (IU/L)**	**50-153**	**192.80**	7.96	5	**200.40**	8.29	5
**LDH (IU/L)**	**0-3062**	792.20	66.21	5	547.20*	49.79	5
**SGOT (IU/L)**	**70-178**	110.40	4.93	5	168.80	72.32	5
**SGPT (IU/L)**	**30-82**	68.60	4.20	5	**96.80**	37.21	5
**Triglycerides (mg/dL)**	**16-77**	58.40	4.83	5	69.40	7.70	5
**CPK (IU/L)**	**0-1595**	1298.60	414.49	5	907.80	188.42	5
**Na (mmol/L)**	**141-148**	141.80	0.49	5	142.20	0.58	5
**K (mmol/L)**	**5.1-6.8**	5.50	0.11	5	5.50	0.13	5
**Chloride (mmol/L)**	**94-104**	101.80	0.80	5	102.20	0.66	5
**GGTP (IU/L)**	**0-1**	0.00	0.00	5	0.00	0.00	5

F= Female; AVG= Average; SEM= Standard Error of the Mean; N= Number

*p<0.05, **p<0.01, ***p<0.001 compared to Vehicle (1F) using T-test

Table S11: Clinical chemistry, Male, Recovery 28 Days Phase

Group	Normal Range	Vehicle (1M)			Allocetra-OTS 1260x10^6 cells/kg (4M)		
		AVG	SEM	N	AVG	SEM	N
**Creatinine (mg/dL)**	**0.27-0.65**	0.29	0.07	5	0.27	0.05	5
**Calcium (mg/dL)**	**9.92-12.28**	11.53	0.15	5	11.55	0.05	5
**Phosphorus(mg/dL)**	**8.1-12.1**	8.40	0.19	5	8.60	0.12	5
**Glucose (mg/dL)**	**50-140**	132.60	2.84	5	134.60	4.12	5
**Urea (mg/dL)**	**29.3-59.2**	37.60	0.93	5	36.70	1.35	5
**Cholesterol (mg/dL)**	**79-137**	**145.00**	7.86	5	132.80	13.92	5
**Total Protein (g/dL)**	**5.92-7.46**	7.01	0.11	5	6.95	0.09	5
**Albumin (g/dL)**	**3.96-4.73**	4.32	0.06	5	4.24	0.05	5
**Globulin (g/dL)**	**1.69-3.01**	2.69	0.11	5	2.71	0.12	5
**Alb/Glob (Ratio)**		1.62	0.07	5	1.58	0.09	5
**Total Bilirubin (mg/dL)**	**0.03-0.18**	0.05	0.01	5	0.06	0.00	5
**Alkaline Phosphatase (IU/L)**	**81-197**	**230.20**	16.21	5	**223.80**	15.59	5
**LDH (IU/L)**	**0-2990**	442.20	73.70	5	331.20	44.46	5
**SGOT (IU/L)**	**57-210**	103.00	6.58	5	99.40	3.63	5
**SGPT (IU/L)**	**30-106**	71.60	2.62	5	75.40	2.94	5
**Triglycerides (mg/dL)**	**21-86**	76.40	5.30	5	81.40	5.14	5
**CPK (IU/L)**	**0-2296**	523.60	144.03	5	458.60	82.52	5
**Na (mmol/L)**	**142-147**	**140.00**	0.55	5	**141.40**	0.93	5
**K (mmol/L)**	**5.3-7.3**	5.70	0.07	5	5.64	0.07	5
**Chloride (mmol/L)**	**94-101**	96.20	1.32	5	96.60	1.69	5
**GGTP (IU/L)**	**0-1**	0.00	0.00	5	0.00	0.00	5

M= Male; AVG= Average; SEM= Standard Error of the Mean; N= Number

*p<0.05, **p<0.01, ***p<0.001 compared to Vehicle (1M) using T-test

Table S12: Clinical chemistry, Female, Recovery 28 Days Phase

Group	Normal Range	Vehicle (1F)			Allocetra-OTS 1260x10^6 cells/kg (4F)		
		AVG	SEM	N	AVG	SEM	N
**Creatinine (mg/dL)**	**0.28-0.65**	0.35	0.01	5	0.36	0.01	5
**Calcium (mg/dL)**	**10.16-12.03**	11.28	0.14	5	11.32	0.10	5
**Phosphorus(mg/dL)**	**7.1-11.6**	8.20	0.43	5	7.70	0.35	5
**Glucose (mg/dL)**	**52-132**	**135.40**	2.42	5	**136.60**	4.57	5
**Urea (mg/dL)**	**28.8-61.3**	38.62	1.13	5	40.28	1.87	5
**Cholesterol (mg/dL)**	**71-148**	114.60	4.40	5	131.40	10.33	5
**Total Protein (g/dL)**	**6-7.31**	6.80	0.17	5	7.00	0.07	5
**Albumin (g/dL)**	**4.20-4.99**	4.56	0.10	5	4.54	0.02	5
**Globulin (g/dL)**	**1.59-2.54**	2.24	0.08	5	2.46	0.09	5
**Alb/Glob (Ratio)**	**-**	2.05	0.06	5	1.86	0.08	5
**Total Bilirubin (mg/dL)**	**0.04-0.21**	0.05	0.01	5	0.06	0.01	5
**Alkaline Phosphatase (IU/L)**	**50-153**	**203.60**	20.69	5	**194.40**	16.27	5
**LDH (IU/L)**	**0-3062**	539.20	68.21	5	545.80	60.20	5
**SGOT (IU/L)**	**70-178**	104.20	5.58	5	104.00	4.79	5
**SGPT (IU/L)**	**30-82**	64.60	0.87	5	73.00	4.16	5
**Triglycerides (mg/dL)**	**16-77**	64.60	2.20	5	55.00	4.36	5
**CPK (IU/L)**	**0-1595**	527.40	84.15	5	**1651.20**	586.67	5
**Na (mmol/L)**	**141-148**	**140.80**	0.20	5	**140.60**	0.24	5
**K (mmol/L)**	**5.1-6.8**	5.50	0.05	5	5.48	0.10	5
**Chloride (mmol/L)**	**94-104**	99.60	0.68	5	99.60	0.68	5
**GGTP (IU/L)**	**0-1**	0.00	0.00	5	0.00	0.00	5

F= Female; AVG= Average; SEM= Standard Error of the Mean; N= Number

*p<0.05, **p<0.01, ***p<0.001 compared to Vehicle (1F) using T-test

Table S13: Coagulation, Males and Females, Main study

Group	Normal Range	Vehicle (1M)			Allocetra-OTS 140x10^6 cells/kg (2M)			Allocetra-OTS 700x10^6 cells/kg (3M)			Allocetra-OTS 1260x10^6 cells/kg (4M)		
		AVG	SEM	N	AVG	SEM	N	AVG	SEM	N	AVG	SEM	N
Fibrinogen (mg/dL)	NA	191.00	14.20	10	201.90	16.14	10	232.90*	12.17	10	277.56**	16.51	10
PT (sec)	10-17.1	10.45	0.18	10	10.45	0.17	10	10.27	0.08	10	10.14	0.05	10
aPTT (sec)	12.2-42.6	14.03	0.68	10	14.18	0.58	10	16.16	0.91	10	15.89*	0.54	10
**Group**	**Normal Range**	**Vehicle (1F)**			**Allocetra-OTS 140x10^6 cells/kg (2F)**			**Allocetra-OTS 700x10^6 cells/kg (3F)**			**Allocetra-OTS 1260x10^6 cells/kg (4F)**		
		**AVG**	**SEM**	**N**	**AVG**	**SEM**	**N**	**AVG**	**SEM**	**N**	**AVG**	**SEM**	**N**
Fibrinogen (mg/dL)	NA	209.4	14.68	10	244.5	8.31	10	232.9	11.12	10	209.6	16.98	10
PT (sec)	10-17.1	10.43	0.19	10	10.22	0.06	10	10.4	0.09	10	10.57	0.18	10
aPTT (sec)	12.2-42.6	15.27	0.63	10	15.89	1.08	10	15.54	0.86	10	14.41	1.35	10

M= Male; F= Female; AVG= Average; SEM= Standard Error of the Mean; N= Number, NA=Not Applicable
*p<0.05, **p<0.01 compared to Vehicle (1M) using T-test

Table S14: Coagulation, Males and Females, Recovery 14 Days Phase

Group	Normal Range	Vehicle (1M)			Allocetra-OTS 1260x10^6 cells/kg (4M)		
		AVG	SEM	N	AVG	SEM	N
Fibrinogen (mg/dL)	NA	257.40	6.70	5	243.00	14.46	5
PT (sec)	10-17.1	10.30	0.03	5	10.38	0.07	5
aPTT (sec)	12.2-42.6	14.80	0.44	5	15.20	0.29	5
**Group**	**Normal Range**	**Vehicle (1F)**			**Allocetra-OTS 1260x10^6 cells/kg (4F)**		
		**AVG**	**SEM**	**N**	**AVG**	**SEM**	**N**
Fibrinogen (mg/dL)	NA	202.80	8.56	5	203.60	13.64	5
PT (sec)	10-17.1	10.34	0.09	5	10.34	0.13	5
aPTT (sec)	12.2-42.6	18.14	0.74	5	16.96	0.65	5

M= Male; F= Female; AVG= Average; SEM= Standard Error of the Mean; N= Number, NA=Not Applicable

Table S15: Coagulation, Males and Females, Recovery 28 Days Phase

Group	Normal Range	Vehicle (1M)			Allocetra-OTS 1260x10^6 cells/kg (4M)		
		AVG	SEM	N	AVG	SEM	N
Fibrinogen (mg/dL)	NA	252.80	13.47	5	239.6	19.48	5
PT (sec)	10-17.1	10.28	0.12	5	10.42	0.17	5
aPTT (sec)	12.2-42.6	15.64	0.46	5	17.62**	0.30	5
**Group**	**Normal Range**	**Vehicle (1F)**			**Allocetra-OTS 1260x10^6 cells/kg (4F)**		
		**AVG**	**SEM**	**N**	**AVG**	**SEM**	**N**
Fibrinogen (mg/dL)	NA	225.6	7.33	5	223.2	17.30	5
PT (sec)	10-17.1	**9.88**	0.04	5	10.06	0.10	5
aPTT (sec)	12.2-42.6	15.08	0.12	5	15.98	0.56	5

M= Male; F= Female; AVG= Average; SEM= Standard Error of the Mean; N= Number; NA=Not Applicable
**p<0.01 compared to Vehicle (1M) using T-test

Table S16: Individual histopathological findings- Male Main Study (Group 1M)

Block No.	Group/Animal<br>Tissue Name	Vehicle (1M)									
		**1**	**2**	**3**	**4**	**5**	**81**	**82**	**83**	**84**	**85**
1	**Heart (LS)**	0	0	0	0	0	0	0	0	0	0
	**Lungs (TS x 2)**		0	0			0	0	0	0	0
	Alveolar histiocytosis	1			1	1					
	Crystals										
2	**Liver (Left and median lobes x2)**		0			0					
	Extramedullary hematopoiesis	1		1	1		1	1	1	1	1
	Hepato-diaphragmatic nodule										2
	**Spleen (TS)**										
	Red pulp - Extramedullary hematopoiesis	1	1	1	1	1	1	1	1	1	1
3	**Brain (TS x 2)**	0	0	0	0	0	0	0	0	0	0
4	**Brain (TS x 3)**	0	0	0	0	0	0	0	0	0	0
5	**Brain (TS x 2)**	0	0	0	0	0	0	0	0	0	0
6	**Kidneys (Left LS x 1, Right TS x1)**	0	0	0			0	0			
	Nephropathy				1	1			1	1	1
	**Urinary bladder**	0	0	0	0	0	0	0	0	0	0
7	**Testes (TS x 2)**	0	0	0	0	0	0	0	0	0	0
8	**Thymus (TS)**	0	0	0	0	0	0	0	0	0	0
9	**Injection Site (Tail TS x 3)**					0					
	Perivascular - hemorrhage	1	2	2	1		2		2	2	2
	Perivascular - inflammation	1	2	2	1		1	1	2	2	1
10	**Aorta (TS)**	0	0	0	0	0	0	0	0	0	0
	**Trachea (TS)**	0	0	0	0	0	0	0	0	0	0
	**Esophagus (TS)**	0	0	0	0	0	0	0	0	0	0
11	**Pancreas**	0	0	0	0	0	0	0	0	0	0
	**Mesenteric Lymph Nodes**	0	0	0	0	0	0	0	0	0	0
	**Mesentery**	0	0	0	0	0	0	0	0	0	0
12	**Stomach (Cardia/ Fundic/ Pylorus LS x 2)**	0	0	0	0	0	0	0	0	0	0
	**Duodenum (TS)**	0	0	0	0	0	0	0	0	0	0
	**Jejunum/ Peyer's Patch (TS)**	0	0	0	0	0	0	0	0	0	0
	**Ileum/ Peyer's Patch (TS)**	0	0	0	0	0	0	0	0	0	0
13	**Caecum (TS)**	0	0	0	0	0	0	0	0	0	0
	**Colon (TS)**	0	0	0	0	0	0	0	0	0	0
	**Rectum (TS)**	0	0	0	0	0	0	0	0	0	0
14	**Males – Skin (LS)**	0	0	0	0	0	0	0	0	0	0
	**Muscle (TS)**	0	0	0	0	0	0	0	0	0	0
15	**Salivary Glands (Mandibular LS x 2)**	0	0	0	0	0	0	0	0	0	0

Block No.	Group/Animal Tissue Name	Vehicle (1M)									
		1	2	3	4	5	81	82	83	84	85
	**Mandibular Lymph Nodes**										
	Lymphoid follicles – increased cellularity	2	2	2	2	2	2	2	2	2	2
16	**Pituitary**	0	0	0	0	0	0	0	0	0	0
17	**Adrenals**	0	0	0	0		0	0	0	0	0
	Accessory nodules					1					
18	**Thyroids**	0	0	0	0	0	0	0	0	0	0
	**Parathyroids**	0	0	0	0	0	0	0	0	0	0
19	**Eyes**	0	0	0	0	0	0	0	0	0	0
	**Optic Nerves (x 2)**	0	0	0	0	0	0	0	0	0	0
20	**Sciatic Nerve (LS x 1, TS x 1)**	0	0	0	0	0	0	0	0	0	0
21	**Spinal Cord (TS x 3)**	0	0	0	0	0	0	0	0	0	0
22	**Epididymis (LS whole x 2)**	0	0	0	0	0	0	0	0	0	0
23	**Seminal Vesicles (TS x 2)**	0	0	0	0	0	0	0	0	0	0
	**Prostate (TS)**	0	0	0	0	0	0	0	0	0	0
24	**Sternum/ Bone Marrow**	0	0	0	0	0	0	0	0	0	0
25	**Femur (femoro-tibial joint)**	0	0	0	0	0	0	0	0	0	0

0 = No Lesion; 1 = Minimal Change; 2 = Mild Change; 3 = Moderate Change; 4 = Marked Change

Table S17: Individual histopathological findings- Male Main study (Group 2M)

Block No.	Group/Animal Tissue Name	Allocetra-OTS 140x10^6 cells /kg (2M)									
		6	7	8	9	10	86	87	88	89	90
2	**Liver (Left and median lobes x2)**			0		0					0
	Extramedullary hematopoiesis	1	1		1		1	1	1	1	
	**Spleen (TS)**										
	Red pulp - Extramedullary hematopoiesis	2	2	2	2	2	2	2	2	2	2
	Red pulp – single cell necrosis (apoptosis)	1	1	2	2	2	2	2	2	2	2

0 = No Lesion; 1 = Minimal Change; 2 = Mild Change; 3 = Moderate Change; 4 = Marked Change

Table S18: Individual histopathological findings- Male Main study (Group 3M)

Block No.	Group/Animal Tissue Name	Allocetra-OTS 700x10^6 cells /kg (3M)									
		21	22	23	24	25	91	92	93	94	95
2	**Liver (Left and median lobes x2)**										
	Extramedullary hematopoiesis	1	1	1	1	1	1	1	1	1	1
	**Spleen (TS)**										
	Red pulp - Extramedullary hematopoiesis	3	3	3	3	3	3	3	3	3	3
	Red pulp – single cell necrosis (apoptosis)	2	2	2	2	2	2	2	2	2	2

0 = No Lesion; 1 = Minimal Change; 2 = Mild Change; 3 = Moderate Change; 4 = Marked Change

Table S19: Individual histopathological findings- Male Main Study (Group 4M)

Block No.	Group/Animal Tissue Name	Allocetra-OTS 1260x10^6 cells /kg (4M)									
		26	27	28	29	30	96	97	98	99	100
1	**Heart (LS)**	0	0	0	0	0	0	0	0	0	0
	**Lungs (TS x 2)**	0	0	0	0	0	0	0	0	0	0
	Alveolar histiocytosis										
	Crystals										
2	**Liver (Left and median lobes x2)**					0					
	Extramedullary hematopoiesis	1	1	1	1		1	1	1	1	1
	Hepato-diaphragmatic nodule		2	2					2		
	**Spleen (TS)**										
	Red pulp - Extramedullary hematopoiesis	3	3	3	3	3	3	3	3	3	3
	Red pulp – single cell necrosis (apoptosis)	2	2	2	2	2	2	2	2	2	2
3	**Brain (TS x 2)**	0	0	0	0	0	0	0	0	0	0
4	**Brain (TS x 3)**	0	0	0	0	0	0	0	0	0	0
5	**Brain (TS x 2)**	0	0	0	0	0	0	0	0	0	0
6	**Kidneys (Left LS x 1, Right TS x1)**	0	0		0		0	0	0	0	
	Nephropathy			1		1					1
	**Urinary bladder**	0	0	0	0	0	0	0	0	0	0
7	**Testes (TS x 2)**	0	0	0	0	0	0	0	0	0	0
8	**Thymus (TS)**	0	0	0	0	0	0	0	0	0	0
9	**Injection Site (Tail TS x 3)**										
	Perivascular - hemorrhage	2	1	1	1	2	1	1	2	2	2
	Perivascular - inflammation	1	2	2	1	1	2	1	2	2	2
10	**Aorta (TS)**	0	0	0	0	0	0	0	0	0	0
	**Trachea (TS)**	0	0	0	0	0	0	0	0	0	0
	**Esophagus (TS)**	0	0	0	0	0	M	0	0	0	0

Block No.	Group/Animal<br>Tissue Name	Allocetra-OTS 1260x10^6 cells /kg (4M)									
		26	27	28	29	30	96	97	98	99	100
11	**Pancreas**	0	0	0	0	0	0	0	0	0	0
	**Mesenteric Lymph Nodes**	0	0	0	0	0	0	0	0	0	0
	**Mesentery**	0	0	0	0	0	0	0	0	0	0
12	**Stomach (Cardia/ Fundic/ Pylorus LS x 2)**	0	0	0	0	0	0	0	0	0	0
	**Duodenum (TS)**	0	0	0	0	0	0	0	0	0	0
	**Jejunum/ Peyer's Patch (TS)**	0	0	0	0	0	0	0	0	0	0
	**Ileum/ Peyer's Patch (TS)**	0	0	0	0	0	0	0	0	0	0
13	**Caecum (TS)**	0	0	0	0	0	0	0	0	0	0
	**Colon (TS)**	0	0	0	0	0	0	0	0	0	0
	**Rectum (TS)**	0	0	0	0	0	0	0	0	0	0
14	**Males – Skin (LS)**	0	0	0	0	0	0	0	0	0	0
	**Muscle (TS)**	0	0	0	0	0	0	0	0	0	0
15	**Salivary Glands (Mandibular LS x 2)**	0	0	0	0	0	0	0	0	0	0
	**Mandibular Lymph Nodes**										
	Lymphoid follicles – increased cellularity	2	2	2	2	2	2	2	2	2	2
16	**Pituitary**	0	0	0	0	0	0	0	0	0	0
17	**Adrenals**	0	0	0	0	0	0	0	0	0	0
18	**Thyroids**	0	0	0	0	0	0	0	0	0	0
	**Parathyroids**	0	0	0	0	0	0	0	0	M	0
19	**Eyes**	0	0	0	0	0	0	0	0	0	0
	**Optic Nerves (x 2)**	0	0	0	0	0	0	0	0	0	0
20	**Sciatic Nerve (LS x 1, TS x 1)**	0	0	0	0	0	0	0	0	0	0
21	**Spinal Cord (TS x 3)**	0	0	0	0	0	0	0	0	0	0
22	**Epididymis (LS whole x 2)**	0	0	0	0	0	0	0	0	0	0
23	**Seminal Vesicles (TS x 2)**	0	0	0	0	0	0	0	0	0	0
	**Prostate (TS)**	0	0	0	0	0	0	0	0	0	0
24	**Sternum/ Bone Marrow**	0	0	0	0	0	0	0	0	0	0
25	**Femur (femoro-tibial joint)**	0	0	0	0	0	0	0	0	0	0

Table S20: Individual histopathological findings- Female Main Study (Group 1F)

Block No.	Group/Animal Tissue Name	Vehicle (1F)									
		51	52	53	54	55	101	102	103	104	105
1	**Heart (LS)**	0	0	0	0	0	0	0	0	0	0
	**Lungs (TS x 2)**	0	0	0	0	0	0	0	0	0	0
	Alveolar histiocytosis										
	Crystals										
2	**Liver (Left and median lobes x2)**		0	0				0	0		0
	Extramedullary hematopoiesis	1			1	1	1			1	1
	Hepato-diaphragmatic nodule										
	**Spleen (TS)**										
	Red pulp - Extramedullary hematopoiesis	1	1	1	1	1	1	1	1	1	1
	Red pulp – single cell necrosis (apoptosis)										
3	**Brain (TS x 2)**	0	0	0	0	0	0	0	0	0	0
4	**Brain (TS x 3)**	0	0	0	0	0	0	0	0	0	0
5	**Brain (TS x 2)**	0	0	0	0	0	0	0	0	0	0
6	**Kidneys (Left LS x 1, Right TS x1)**	0	0	0	0		0	0	0	0	0
	Nephropathy					1					
	**Urinary bladder**	0	0	0	0	0	0	0	0	0	0
7	**Ovaries (x2)**	0	0	0	0	0	0	0	0	0	0
	**Oviducts (LS x 2)**	0	0	0	0	0	0	0	0	0	0
8	**Thymus (TS)**	0	0	0	0	0	0	0	0	0	0
9	**Injection Site (Tail TS x 3)**										
	Perivascular - hemorrhage	2	2	2	2	2	2	2	2	2	1
	Perivascular - inflammation	1	1	1		2	2	2	2	2	1
10	**Aorta (TS)**	0	0	0	0	0	0	0	0	0	0
	**Trachea (TS)**	0	0	0	0	0	0	0	0	0	0
	**Esophagus (TS)**	0	0	0	0	0	0	0	M	0	0
11	**Pancreas**	0	0	0	0	0	0	0	0	0	0
	**Mesenteric Lymph Nodes/ Mesentery**	0	0	0	0	0	0	0	0	0	0
12	**Stomach (Cardia/ Fundic/ Pylorus LS x 2)**	0	0	0	0	0	0	0	0	0	0
	**Duodenum (TS)**	0	0	0	0	0	0	0	0	0	0
	**Jejunum/ Peyer's Patch (TS)**	0	0	0	0	0	0	0	0	0	0
	**Ileum/ Peyer's Patch (TS)**	0	0	0	0	0	0	0	0	0	0
13	**Caecum (TS)**	0	0	0	0	0	0	0	0	0	0
	**Colon (TS)**	0	0	0	0	0	0	0	0	0	0
	**Rectum (TS)**	0	0	0	0	0	0	0	0	0	0
14	**Skin/ Mammary Gland (LS)**	0	0	0	0	0	0	0	0	0	0

Block No.	Group/Animal Tissue Name	Vehicle (1F)									
		51	52	53	54	55	101	102	103	104	105
	**Muscle (TS)**	0	0	0	0	0	0	0	0	0	0
15	**Salivary Glands (Mandibular LS x 2)**	0	0	0	0	0	0	0	0	0	0
	**Mandibular Lymph Nodes**										
	Lymphoid follicles – increased cellularity	2	2	2	2	2	2	1	2	2	2
16	**Pituitary**	0	0	0	0	0	0	0	0	0	0
17	**Adrenals**	0			0	0	0	0	0	0	0
	Accessory nodules		1	1							
18	**Thyroids**	0	0	0	0	0	0	0	0	0	0
	**Parathyroids**	0	0	0	0	0	0	M	0	0	0
19	**Eyes (x 2)**	0	0	0	0	0	0	0	0	0	0
	**Optic Nerves (x 2)**	0	0	0	0	0	0	0	0	0	0
20	**Sciatic Nerve (LS x 1, TS x 1)**	0	0	0	0	0	0	0	0	0	0
21	**Spinal Cord (TS x 3)**	0	0	0	0	0	0	0	0	0	0
22	**Uterus/ Cervix/ Vagina (LS x 1, TS x 2)**	0	0	0	0	0	0	0	0	0	0
24	**Sternum/ Bone Marrow**	0	0	0	0	0	0	0	0	0	0
25	**Femur (femoro-tibial joint)**	0	0	0	0	0	0	0	0	0	0

0 = No Lesion; 1 = Minimal Change; 2 = Mild Change; 3 = Moderate Change; 4 = Marked Change; M=Missing

Table S21: Individual histopathological findings- Female Main study (Group 2F)

Block No.	Group/Animal Tissue Name	Allocetra-OTS 140x10^6 cells /kg (2F)									
		56	57	58	59	60	106	107	108	109	110
2	**Liver (Left and median lobes x2)**						0				
	Extramedullary hematopoiesis	1	1	1	1	1		1	1	1	1
	**Spleen (TS)**										
	Red pulp - Extramedullary hematopoiesis	2	2	2	2	2	2	2	2	2	2
	Red pulp – single cell necrosis (apoptosis)	2	2	2	2	2	2	2	2	2	2

0 = No Lesion; 1 = Minimal Change; 2 = Mild Change; 3 = Moderate Change; 4 = Marked Change

Table S22: Individual histopathological findings- Female Main study (Group 3F)

Block No.	Group/Animal Tissue Name	Allocetra-OTS 700x10^6 cells /kg (3F)									
		71	72	73	74	75	111	112	113	114	115
2	**Liver (Left and median lobes x2)**										
	Extramedullary hematopoiesis	1	1	1	1	1	1	1	1	1	1
	**Spleen (TS)**										

Block No.	Group/Animal Tissue Name	Allocetra-OTS 700x10^6 cells /kg (3F)									
		71	72	73	74	75	111	112	113	114	115
	Red pulp - Extramedullary hematopoiesis	3	3	3	3	3	3	3	3	3	3
	Red pulp – single cell necrosis (apoptosis)	2	2	2	2	2	2	2	2	2	2

0 = No Lesion; 1 = Minimal Change; 2 = Mild Change; 3 = Moderate Change; 4 = Marked Change

Table S23: Individual histopathological findings- Female Main Study (Group 4F)

Block No.	Group/Animal Tissue Name	Allocetra-OTS 1260x10^6 cells /kg (4F)									
		76	77	78	79	80	116	117	118	119	120
1	**Heart (LS)**	0	0	0	0	0	0	0	0	0	0
	**Lungs (TS x 2)**	0	0	0	0	0	0	0		0	0
	Alveolar histiocytosis								1		
	Crystals								1		
2	**Liver (Left and median lobes x2)**		0	0			0				
	Extramedullary hematopoiesis	2			1	1		1	1	1	1
	Hepato-diaphragmatic nodule										
	**Spleen (TS)**										
	Red pulp - Extramedullary hematopoiesis	2	3	3	3	3	3	3	3	3	3
	Red pulp – single cell necrosis (apoptosis)	1	2	2	2	2	2	2	2	2	2
3	**Brain (TS x 2)**	0	0	0	0	0	0	0	0	0	0
4	**Brain (TS x 3)**	0	0	0	0	0	0	0	0	0	0
5	**Brain (TS x 2)**	0	0	0	0	0	0	0	0	0	0
6	**Kidneys (Left LS x 1, Right TS x1)**	0	0	0	0	0	0	0			
	Nephropathy								1	1	1
	**Urinary bladder**	0	0	0	0	0	0	0	0	0	0
7	**Ovaries (x2)**	0	0	0	0	0	0	0	0	0	0
	**Oviducts (LS x 2)**	0	0	0	0	0	0	0	0	0	0
8	**Thymus (TS)**	0	0	0	0	0	0	0	0	0	0
9	**Injection Site (Tail TS x 3)**							0			
	Perivascular - hemorrhage	2	2	2	2		2		2	2	2
	Perivascular - inflammation	2	1	2	2	1	1		2	1	2
10	**Aorta (TS)**	0	0	0	0	0	0	0	0	0	0
	**Trachea (TS)**	0	0	0	0	0	0	0	0	0	0
	**Esophagus (TS)**	0	0	0	0	0	0	0	M	0	0
11	**Pancreas**	0	0	0	0	0	0	0	0	0	0
	**Mesenteric Lymph Nodes/ Mesentery**	0	0	0	0	0	0	0	0	0	0
12	**Stomach (Cardia/ Fundic/ Pylorus LS x 2)**	0	0	0	0	0	0	0	0	0	0
	**Duodenum (TS)**	0	0	0	0	0	0	0	0	0	0

Block No.	Group/Animal Tissue Name	Allocetra-OTS 1260x10^6 cells /kg (4F)									
		76	77	78	79	80	116	117	118	119	120
	**Jejunum/ Peyer's Patch (TS)**	0	0	0	0	0	0	0	0	0	0
	**Ileum/ Peyer's Patch (TS)**	0	0	0	0	0	0	0	0	0	0
13	**Caecum (TS)**	0	0	0	0	0	0	0	0	0	0
	**Colon (TS)**	0	0	0	0	0	0	0	0	0	0
	**Rectum (TS)**	0	0	0	0	0	0	0	0	0	0
14	**Skin/ Mammary Gland (LS)**	0	0	0	0	0	0	0	0	0	0
	**Muscle (TS)**	0	0	0	0	0	0	0	0	0	0
15	**Salivary Glands (Mandibular LS x 2)**	0	0	0	0	0	0	0	0	0	0
	**Mandibular Lymph Nodes**										
	Lymphoid follicles – increased cellularity	2	2	2	2	2	2		2	2	2
	Congestion							2			
16	**Pituitary**	0	0	0	0	0	0	0	0	0	0
17	**Adrenals**	0	0	0	0	0	0	0	0	0	
	Accessory nodules										1
18	**Thyroids**	0	0	0	0	0	0	0	0	0	0
	**Parathyroids**	0	0	0	0	0	0	0	0	0	0
19	**Eyes (x 2)**	0	0	0	0	0	0	0	0	0	0
	**Optic Nerves (x 2)**	0	0	0	0	0	0	0	0	0	0
20	**Sciatic Nerve (LS x 1, TS x 1)**	0	0	0	0	0	0	0	0	0	0
21	**Spinal Cord (TS x 3)**	0	0	0	0	0	0	0	0	0	0
22	**Uterus/ Cervix/ Vagina (LS x 1, TS x 2)**	0	0	0	0	0	0	0	0	0	0
24	**Sternum/ Bone Marrow**	0	0	0	0	0	0	0	0	0	0
25	**Femur (femoro-tibial joint)**	0	0	0	0	0	0	0	0	0	0

0 = No Lesion; 1 = Minimal Change; 2 = Mild Change; 3 = Moderate Change; 4 = Marked Change; M=Missing

Table S24: Individual histopathological findings- 14 days Recovery Phase, Groups 1M and 4M

Block No.	Group/Animal Tissue Name	Vehicle (1M)					Allocetra-OTS 1260x10^6 cells /kg (4M)				
		41	42	43	44	45	46	47	48	49	50
1	**Heart (LS)**	0	0	0	0	0	0	0	0		0
	Fibrosis									1	
	**Lungs (TS x 2)**	0	0	0	0	0	0	0	0	0	0
2	**Liver (Left and median lobes x2)**							0		0	0
	Extramedullary hematopoiesis	1	1	1	1	1	1		1		
	**Spleen (TS)**										
	Red pulp - Extramedullary hematopoiesis	1	1	1	1	1	1	1	1	1	1

Block No.	Group/Animal Tissue Name	Vehicle (1M)					Allocetra-OTS 1260x10^{6} cells /kg (4M)				
		41	42	43	44	45	46	47	48	49	50
	Red pulp – single cell necrosis (apoptosis)										
3	**Brain (TS x 2)**	0	0	0	0	0	0	0	0	0	0
4	**Brain (TS x 3)**	0	0	0	0	0	0	0	0	0	0
5	**Brain (TS x 2)**	0	0	0	0	0	0	0	0	0	0
6	**Kidneys (Left LS x 1, Right TS x1)**		0	0		0	0	0		0	0
	Nephropathy	1			1				1		
7	**Testes (TS x 2)**	0	0	0	0	0	0	0	0	0	0
8	**Thymus (TS)**	0	0	0	0	0	0	0	0	0	0
9	**Injection Site (Tail TS x 3)**		0	0	0	0	0	0	0		0
	Crust	1								1	

0 = No Lesion; 1 = Minimal Change; 2 = Mild Change; 3 = Moderate Change; 4 = Marked Change

Table S25: Individual histopathological findings- 14 days Recovery Phase, Groups 1F and 4F

Block No.	Group/Animal Tissue Name	Vehicle (1F)					Allocetra-OTS 1260x10^{6} cells /kg (4F)				
		11	12	13	14	15	16	17	18	19	20
1	**Heart (LS)**	0	0	0	0	0	0	0	0	0	0
	**Lungs (TS x 2)**	0	0	0	0	0	0	0	0		0
	Alveolar histiocytosis									1	
2	**Liver (Left and median lobes x2)**				0		0	0			
	Extramedullary hematopoiesis	1	1	1		1			1	1	1
	**Spleen (TS)**										
	Red pulp - Extramedullary hematopoiesis	1	1	1	1	1	1	1	1	1	1
	Red pulp – single cell necrosis (apoptosis)										
3	**Brain (TS x 2)**	0	0	0	0	0	0	0	0	0	0
4	**Brain (TS x 3)**	0	0	0	0	0	0	0	0	0	0
5	**Brain (TS x 2)**	0	0	0	0	0	0	0	0	0	0
6	**Kidneys (Left LS x 1, Right TS x1)**	0	0	0	0	0	0	0			0
	Nephropathy								1	1	
7	**Ovaries (x2)**	0	0	0	0	0	0	0	0	0	0
	**Oviducts (LS x 2)**	0	0	0	0	0	0	0	0	0	0
8	**Thymus (TS)**	0	0	0	0	0	0	0	0	0	0
9	**Injection Site (Tail TS x 3)**	0	0	0	0	0	0	0	0	0	0
	Crust										

0 = No Lesion; 1 = Minimal Change; 2 = Mild Change; 3 = Moderate Change; 4 = Marked Change

Table S26: Individual histopathological findings- 28 days Recovery phase, Group 1M and 4M

Block No.	Group/Animal Tissue Name	Vehicle (1M)					Allocetra-OTS 1260x10^6 cells /kg (4M)				
		61	62	63	64	65	66	67	68	69	70
2	**Liver (Left and median lobes x2)**	0								0	
	Extramedullary hematopoiesis		1	1	1	1	1	1	1		1
	**Spleen (TS)**										
	Red pulp - Extramedullary hematopoiesis	1	1	1	1	1	1	1	1	1	1
	Red pulp – single cell necrosis (apoptosis)										

0 = No Lesion; 1 = Minimal Change; 2 = Mild Change; 3 = Moderate Change; 4 = Marked Change

Table S27: Individual histopathological findings- 28 days Recovery phase, Group 1F and 4F

Block No.	Group/Animal Tissue Name	Vehicle (1F)					Allocetra-OTS 1260x10^6 cells /kg (4F)				
		31	32	33	34	35	36	37	38	39	40
2	**Liver (Left and median lobes x2)**										
	Extramedullary hematopoiesis	1	1	1	1	1	1	1	1	1	1
	**Spleen (TS)**										
	Red pulp - Extramedullary hematopoiesis	1	1	1	1	1	1	1	1	1	1
	Red pulp – single cell necrosis (apoptosis)										

0 = No Lesion; 1 = Minimal Change; 2 = Mild Change; 3 = Moderate Change; 4 = Marked Change
